# Gill Transcriptomic Responses to Toxin-producing Alga *Prymnesium parvum* in Rainbow Trout

**DOI:** 10.3389/fimmu.2021.794593

**Published:** 2021-12-08

**Authors:** Morag Clinton, Elżbieta Król, Dagoberto Sepúlveda, Nikolaj R. Andersen, Andrew S. Brierley, David E. K. Ferrier, Per Juel Hansen, Niels Lorenzen, Samuel A. M. Martin

**Affiliations:** ^1^School of Biological Sciences, University of Aberdeen, Aberdeen, United Kingdom; ^2^Scottish Oceans Institute, University of St Andrews, St Andrews, United Kingdom; ^3^Department of Veterinary Medicine, University of Alaska Fairbanks, Fairbanks, AK, United States; ^4^National Institute of Aquatic Resources, Technical University of Denmark, Kgs. Lyngby, Denmark; ^5^ Department of Bioscience, Aarhus University, Roskilde, Denmark; ^6^Department of Biology, Marine Biological Section, University of Copenhagen, Helsingør, Denmark

**Keywords:** gill health, gill inflammation, microarray, cytokines, hypoxia, harmful algal bloom (HAB), golden alga

## Abstract

The gill of teleost fish is a multifunctional organ involved in many physiological processes, including protection of the mucosal gill surface against pathogens and other environmental antigens by the gill-associated lymphoid tissue (GIALT). Climate change associated phenomena, such as increasing frequency and magnitude of harmful algal blooms (HABs) put extra strain on gill function, contributing to enhanced fish mortality and fish kills. However, the molecular basis of the HAB-induced gill injury remains largely unknown due to the lack of high-throughput transcriptomic studies performed on teleost fish in laboratory conditions. We used juvenile rainbow trout (*Oncorhynchus mykiss*) to investigate the transcriptomic responses of the gill tissue to two (high and low) sublethal densities of the toxin-producing alga *Prymnesium parvum*, in relation to non-exposed control fish. The exposure time to *P. parvum* (4–5 h) was sufficient to identify three different phenotypic responses among the exposed fish, enabling us to focus on the common gill transcriptomic responses to *P. parvum* that were independent of dose and phenotype. The inspection of common differentially expressed genes (DEGs), canonical pathways, upstream regulators and downstream effects pointed towards *P. parvum*-induced inflammatory response and gill inflammation driven by alterations of Acute Phase Response Signalling, IL-6 Signalling, IL-10 Signalling, Role of PKR in Interferon Induction and Antiviral Response, IL-8 Signalling and IL-17 Signalling pathways. While we could not determine if the inferred gill inflammation was progressing or resolving, our study clearly suggests that *P. parvum* blooms may contribute to the serious gill disorders in fish. By providing insights into the gill transcriptomic responses to toxin-producing *P. parvum* in teleost fish, our research opens new avenues for investigating how to monitor and mitigate toxicity of HABs before they become lethal.

## Introduction

The gill of teleost fish is a complex organ whose function goes beyond extracting oxygen from water and excreting carbon dioxide. Apart from the respiratory gas exchange, the gill plays a key role in osmotic and ionic regulation, acid-base balance, and excretion of nitrogenous waste (1). Because these processes are predominantly surface-dependent, the gill tissue consists of a highly complex system of branching vascular structures (primary and secondary lamellae) that are separated from the environment only by a thin layer of gill epithelium and mucosa (2, 3). As a result, the epithelial surface area of the gill is typically larger than the total surface of skin (4). A substantial complexity of the teleost gill has also been demonstrated at the cellular resolution, with recent identification of 20 distinct cell clusters in the gill of Atlantic salmon (*Salmo salar*), using a single-nuclei RNA-seq approach (5).

Having the large epithelial surface of the gill system open to the external milieu comes with some disadvantages. Among them is the risk of mechanical injuries and gill abrasion, which may cause haemorrhage and contribute to gill inflammation (6). Furthermore, the large surface area of the gill may facilitate the uptake of toxic substances, including those occurring naturally (e.g., algal toxins, metal ions and ammonia) and the whole range of man-made pollutants (e.g., industrial chemicals, pesticides and microplastics) (7–9). Last, but not least, the gill surface provides major ports of entry for pathogens (via transepithelial transport) or site of interaction with other harmful invertebrates, such as water-born parasites and cnidarian jellyfish (10). To meet these challenges, the gill is strategically equipped with its own immune system, called the gill-associated lymphoid tissue (GIALT), thereby substantially contributing to overall fish health and survival (2, 3). The GIALT consists of a panel of resident immune cells, including B and T cells, monocytes, macrophages, neutrophils, thrombocytes, dendritic-like cells, natural killer-like cells, eosinophilic granule cells, rodlet cells, and melanin-containing cells among the others (11, 12).

Evidence is growing that climate change is putting extra strain on gill health and performance in many environments, including aquaculture (13). Rising water temperatures elevate metabolism of fish and their demands for oxygen (14), but at the same time decrease the oxygen content of water (15), intensifying the osmorespiratory conflict between the functionally large gill surface area (to promote respiratory gas exchange) and the need for the reduced gill surface area (to limit water and ion fluxes) (16, 17). To avoid unfavourable changes in the environment, many marine organisms (including pathogens and parasites) are shifting their distributions as ocean temperatures warm (18). Among the hallmarks of the rapidly changing aquatic ecosystems are the occurrences of harmful algal blooms (HABs), whose increasing frequency, magnitude, and duration as well as migration poleward have been linked to ocean warming, marine heat waves, oxygen loss, eutrophication, and pollution (19, 20). The climate-related range shifts and expansion in marine and freshwater environments put many species of fish at risk of being exposed to a variety of novel algal toxins, pathogens, and parasites, which were never before part of their own evolutionary history (21). While wild fish can relocate, farmed fish are heavily restricted in their movements, which makes their gills particularly vulnerable to the environmental insults. Thus, investigating the link between the climate change phenomena and gill health has wide implications not only for biodiversity and sustainability of aquatic ecosystems but also for food security at the global scale (13).

One of the HAB species that is particularly toxic to fish and other gill-breathing animals is the haptophyte alga *Prymnesium parvum*, undergoing a rapid range expansion in coastal and inland waters worldwide. Commonly referred to as the golden alga, *P. parvum* has been documented to kill ~135 metric tons of farmed Atlantic salmon (*Salmo salar*) in Norway during a bloom in 2007 (22). In inland water bodies of Texas, ~34 million fish valued at ~13 million US dollars were lost due to *P. parvum*-related fish kills between 1981 and 2008 (23). As proposed decades ago (24), there is consensus that *P. parvum* acts on the gill tissue, but the exact mechanisms of its action are under investigation. Some research suggests that *P. parvum* cells need to be in direct physical contact with the gill surface to release harmful toxins, as a part of the toxin-assisted micropredation (25, 26). Others propose that the presence of *P. parvum* toxins in the water is sufficient to kill fish (27, 28). Although the complete suite of potential toxic compounds produced by *P. parvum* may not have been fully characterized, increasing evidence points towards prymnesins (a class of ladder-frame polyether phycotoxins) being responsible for enhanced fish mortality and massive fish kills (29–31). Yet, the experimental manipulations of fish with the use of toxin-producing *P. parvum* are relatively scarce.

Some studies use fish only for testing the acute toxicity of *P. parvum* cultures to establish their LC_50_ values (mortality assay), with little focus on the fish themselves (28, 32–37). Likewise, red cells extracted from fish blood have been used *ex vivo* to evaluate the haemolytic activity (a proxy for toxicity) of *P. parvum* isolates (38, 39). In contrast, the sublethal doses of *P. parvum* were employed to investigate the effects of HABs on the whole-animal respiratory physiology (e.g., oxygen consumption, ventilation volume and frequency) in rainbow trout (*Oncorhynchus mykiss*) and European plaice (*Pleuronectes platessa*), with both studies pointing towards substantially reduced capacity of gills to extract oxygen from the environment (8, 40). Both fish larvae (fathead minnow *Pimephales promelas* and zebrafish *Danio rerio*) as well as fish liver (Hepa-E1 and PLHC-1) and gill (G1B and RTgill-W1) cell lines were exposed to the sublethal doses of *P. parvum* to explore various aspects of the toxin-induced oxidative stress and antioxidant defence, including lipid peroxidation, oxidative DNA damage as well as gene expression and activity of antioxidant enzymes (41–43). It has also been demonstrated that the sublethal exposure of rainbow trout to *P. parvum* modulates susceptibility of fish to infectious agents such as viral haemorrhagic septicaemia virus (VHSV) (44). Surprisingly, little research has been done on the gill transcriptome, despite the gill being considered a key target tissue for *P. parvum* action.

In the current study, we used juvenile rainbow trout to investigate the transcriptomic responses of the gill tissue to two (high and low) sublethal densities of toxin-producing *P. parvum*, in relation to non-exposed control fish. The exposure time to *P. parvum* (4–5 h) was sufficient to identify three different phenotypic responses among the exposed fish, enabling us to focus on the common gill transcriptomic responses to *P. parvum* that were independent of dose and phenotype. Instead of profiling individual genes, we performed a microarray experiment to evaluate the gene expression changes at the level of whole tissue transcriptome (45). The functional analysis of the differentially expressed genes (DEGs) induced by *P. parvum* exposure included identification of associated canonical pathways, upstream regulators, and downstream effects.

## Materials and Methods

### Fish and Housing

All animal work was performed at the University of Aarhus (Denmark) in summer 2016. We used juvenile females of outbred rainbow trout (*Oncorhynchus mykiss*, body mass 10–15 g) from an all-female stock, hatched and reared in fresh water (temperature 10–11°C) under pathogen-free laboratory conditions. Fish were allocated to 8 experimental tanks (16 fish per 10-L tank, water temperature 15–17°C, water salinity 1.1%, water oxygen saturation > 90%) and then allowed to acclimate to the experimental conditions for 5 days. During the acclimation and experiment, fish were fed a standard commercial diet.

### Exposure to *Prymnesium parvum*

The haptophyte *P. parvum* (Kalmar University Culture Collection, strain KAC 39) were cultured in F/2 medium (temperature 15°C, salinity 0.9%, photoperiod 14 h:10 h light:dark) as described previously (44). The exposure of fish to *P. parvum* was performed by adding exponentially growing cultures of *P. parvum* to the water to create environments with high (~4 x 10^4^ cells per mL of water) and low (~1.5 x 10^4^ cells per mL of water) densities of algae. During the transfer, the viability of the algal cells was confirmed by microscopy, after which the cells were pipetted into the water column and gently mixed to ensure their homogenous distribution within the tank. The *P. parvum* densities were chosen to mimic natural blooms (46), with both doses expected to have sublethal effects on fish (44). The effects of high and low doses of algae were evaluated using 3 replicate tanks. The remaining 2 tanks had no *P. parvum* added and served as negative controls.

### Fish Sampling

Immediately after the exposure to *P. parvum*, fish were closely and continuously monitored for any behavioral and physiological abnormalities until the end of experiment (5 h post-exposure). This led to the identification of three different phenotypic responses that gradually developed among the exposed fish, referred to as high response, moderate response and low response (for details see **Table 1**). The high and moderate phenotypic responses were expressed by fish exposed to the high dose of *P. parvum*, while low phenotypic responses were observed in fish exposed to the low dose of *P. parvum*. Based on the dose and phenotype, fish were classified into 4 groups with high exposure/high response (HH), high exposure/moderate response (HM), low exposure/low response (LL) and control group (C) with no exposure/no response (**Table 1**).

**Table 1:** Experimental setup and classification of rainbow trout into groups based on the level of exposure to the toxin-producing alga *Prymnesium parvum* (high and low) and the fish phenotypic response to the algae (high, moderate and low).

Tank	Exposure to *P. parvum*	Fish phenotypic response (clinical presentation)	Group
1–3	High^1^	High response: increased respiratory effort, advanced lethargy, loss of balance, dark skin colour, increased production of gill mucus	HH (high exposure/high response)
		Moderate response: increased respiratory effort, mild lethargy, increased production of gill mucus	HM (high exposure/moderate response)
4–6	Low^2^	Low response: increased production of gill mucus	LL (low exposure/low response)
7, 8	None	None	C (control, no exposure/no response)

^1^~4 x 10^4^ cells per mL of water, ^2^~1.5 x 10^4^ cells per mL of water.

The resultant groups consisted of 16 fish each (64 fish in total).

Control fish (n = 16) were sampled 3–4 h after starting the experiment, followed by the experimental fish (n = 48), which were sampled 4–5 h after the exposure to *P. parvum*. Sampling was alternated between the HH, HM and LL groups for experimental balance. The experiment was terminated at 5 h post-exposure to reduce the risk of fish suffering from the algal toxicity (44).

Before sampling, fish were first euthanized by immersion in 0.01% benzocaine, then bled by removing the caudal fin and finally subjected to excision of gill arches. The collected gill tissue (2 separate gill arches per fish) was quickly blotted with absorbing paper to remove residual blood, followed by immediate transfer to RNAlater^®^ (Sigma-Aldrich, St. Louis, MO, United States). The gill samples (8 gill arches from 4 fish from the same group per tube) were kept at 4°C overnight for equilibration and then stored at −20°C prior to RNA extraction.

### RNA Extraction and Sample Pooling

Total RNA extraction was performed on individual gill samples (n = 64), including the gill arch and full-length filaments. The RNA was isolated by homogenization of ~100 mg of gill tissue in TRIzol^®^ Reagent (Ambion by Life Technologies, Carlsbad, CA, United States), using 3 mm tungsten carbide beads and a TissueLyser II Disruption System (Qiagen GmbH, Hilden, Germany). Afterwards, the RNA was quantified by spectrophotometry (NanoDrop Technologies, Wilmington, DE, United States), with the RNA integrity assessed by electrophoresis (Agilent Technologies, Santa Clara, CA, United States). The individual RNA samples were subsequently pooled to generate 4 biological replicates per each group, with an equimolar contribution of RNA from 4 gill samples to each pool, yielding 16 RNA pools in total (4 groups x 4 RNA pools x 4 gill samples per pool). The 4 gill samples per pool originated from 2–4 fish.

### Microarray Experiment

We used a custom designed Agilent oligonucleotide microarray platform Trout_imm_v1 (Agilent design ID: 028918) with 4 x 44 K probes per slide, developed for rainbow trout and validated using RTqPCR (47). The experiment consisted of 16 hybridisations (4 groups × 4 RNA pools), which were performed on 4 microarray slides in a semi-randomised order (each slide with 4 different groups). All RNA pools (n = 16) were subsampled to generate a common control, with contribution of the gill total RNA from 32–64 fish (4 groups × 4 RNA pools x 2–4 fish per pool).

RNA amplification and labelling, followed by microarray hybridisation, scanning and feature extraction were performed as described previously (47–49). Briefly, antisense amplified RNA (aRNA) was generated from ~2 μg total RNA per experimental or common control pool, using Amino Allyl MessageAmp™ II aRNA Amplification Kit (Ambion by Life Technologies, Carlsbad, CA, USA). Pools of aRNA were then coupled with amine reactive Cy fluorescent dyes (Amersham™ Cy™3 and Cy™5 Mono-reactive Dye Packs; GE Healthcare UK Limited, Little Chalfont, UK). All experimental samples were labelled with Cy3, while Cy5 was used to label the common control. The reaction products were purified using a DyeEx^®^ 2.0 Spin Kit (Qiagen GmbH, Hilden, Germany). Dye incorporation and post-labelling aRNA yield were quantified by spectrophotometry (NanoDrop Technologies, Wilmington, DE, USA). Each hybridisation was performed using 825 ng of Cy3-labelled experimental sample and 825 ng of Cy5-labelled common control. The aRNA was first fragmented and then hybridised at 65°C for 17 h in an Agilent hybridisation oven. Following hybridisation, slides were subjected to washing steps, after which they were air dried in the dark and scanned within 2 h. Scanning was carried out at 5 μm resolution on a Gene-Pix Personal 4100A scanner (Axon Instruments, Molecular Devices Corp., Sunnyvale, CA, USA), with the PMT values adjusted manually to ensure the mean intensity ratio of Cy3:Cy5 signal was close to 1. Agilent Feature Extraction Software (version 9.5.3) was used to identify features and to extract the raw intensity values for subsequent statistical analysis.

### Microarray Data Analysis

Feature intensities were pre-processed and analysed for differential gene expression using the Bioconductor package limma (50) in R v3.5.0 (51). Briefly, loess normalisation was performed within each array to account for intensity-dependent variation in dye bias along with quantile normalisation used to stabilize experimental variances across arrays. Normalised data were subsequently filtered to remove control features and non-responsive RNA targets with equal expression across 4 groups. Differential expression of RNA targets between groups was assessed using linear modelling, with the contrasts set up to compare each group of the fish exposed to *P. parvum* with the non-exposed control group (i.e., HH vs C, HM vs C and LL vs C). Multiple testing was accounted for by controlling the false discovery rate at 5% using the Benjamini-Hochberg procedure. The RNA targets with adjusted p-value < 0.05 and absolute Log_2_ FC > 1 were considered as differentially expressed. All RNA targets meeting these criteria were 1) checked for the number of unique fish genes (with approximately half of the probes being redundant) and 2) mapped to human orthologs to enhance the functional analysis of gene expression (**Table 2**, **Supplementary Tables 1**–**3**).

**Table 2:** Results of differential gene expression analysis performed on the gill transcriptome of rainbow trout exposed to the toxin-producing alga *Prymnesium parvum*.

Group comparison	Number of differentially expressed genes^1^			Supplementary Table
	RNA targets^2^	Fish genes	HGNC gene ID	
HH vs control	3107	1519	1436	1
HM vs control	2348	1177	1069	2
LL vs control	1247	606	567	3

^1^RNA targets (representing fish mRNAs bound to the microarray probes) were mapped to human orthologs (BLASTX, E-value < 0.00001, top hit) to generate HGNC gene identifiers (HGNC, HUGO Gene Nomenclature Committee). Numbers refer to the total number of RNA targets (including replicate and redundant probes), and to the unique numbers of fish genes and human orthologs mapped to these targets.

^2^for volcano plots of differential expression of RNA targets see **Supplementary Figure 3**.

The contrasts were set up to compare fish from HH (high exposure/high response), HM (high exposure/moderate response) and LL (low exposure/low response) groups vs control group (no exposure/no response). Genes were considered differentially expressed at adjusted p-value < 0.05 and absolute Log_2_ FC > 1.

### Fish-to-Human Orthologs

Differentially expressed RNA targets were mapped to human orthologs to generate HGNC (HUGO Gene Nomenclature Committee) gene identifiers needed for functional analysis. This approach was demonstrated to enhance functional analysis of fish genes by providing access to well-annotated databases and tools for mammalian model organisms (mice, rats, and humans), despite differences between fish and mammals in gene function and molecular pathways (49, 52, 53). Mapping was done by aligning the microarray probe sequences (associated with the differentially expressed RNA targets) to the protein sequences from the human genome (release 88, downloaded from Ensembl at https://www.ensembl.org/Homo_sapiens/Info/Index) using BLASTX (version 2.2.31) (54) with an E-value cut off of 0.00001 and a maximum of 1 human gene for each probe (top hit). Although most of the RNA targets mapped to the unique human orthologs (1 RNA target → 1 unique HGNC gene identifier), some human orthologs were associated with multiple RNA targets. If the multiple RNA targets mapped to the same HGNC gene had the same direction of change (either upregulated or downregulated), their expression values were averaged to provide a mean Log_2_ FC for the fish-to-human ortholog. The multiple RNA targets with the opposite direction of change (both upregulated and downregulated) were excluded from the functional analysis of gene expression (for details see **Supplementary Tables 1**–**3**).

### Functional Analysis of Gene Expression

Human orthologs of the differentially expressed fish genes were analysed using Ingenuity Pathway Analysis (IPA, QIAGEN Redwood City, www.qiagen.com/ingenuity). In total, 3 sets of differentially expressed genes (DEGs) were submitted to IPA (along with their Log_2_ FC values), representing 3 comparisons between the fish groups: HH vs control (1436 DEGs), HM vs control (1069 DEGs) and LL vs control (567 DEGs) (**Table 2**, **Supplementary Tables 1**–**3**). These gene sets were analysed using the Ingenuity Knowledge Base (genes only) as a reference set, including in the analysis all species (mice, rats, and humans) as well as all tissues and cell lines (default settings). The main focus of the functional analysis were canonical pathways, upstream regulators and downstream effects, the significance of which was based on the Benjamini-Hochberg (B-H) multiple testing correction p-value, with the overall activation/inhibition states predicted by the IPA z-score algorithm (z-score ≥ 2 predicts an increase in activity while z-score ≤ −2 predicts a decrease in activity). Gene ratios for each canonical pathway were calculated as the number of DEGs contributing to the pathway divided by the total number of genes that constitute the pathway and are present in the reference set.

## Results

### Differentially Expressed Genes (DEGs)

**Figure 1:**
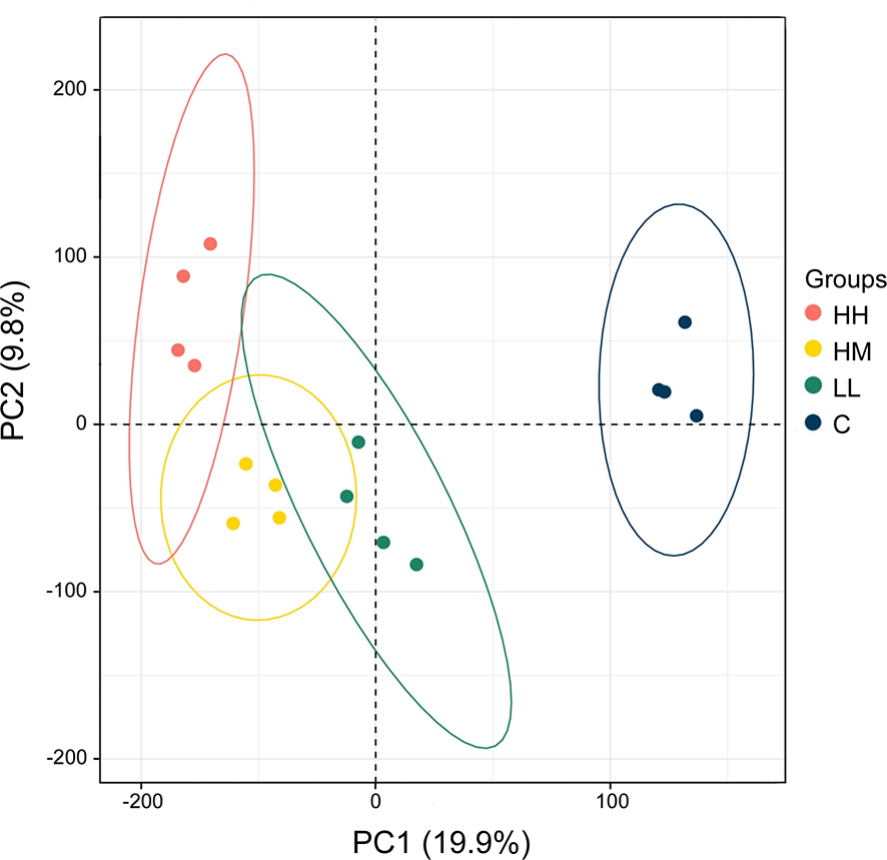
Principal component analysis (PCA) of the gill transcriptome profiles in rainbow trout from HH (high exposure/high response), HM (high exposure/moderate response), LL (low exposure/low response) and C (control, no exposure/no response) groups. Each circle refers to all genes from one microarray hybridisation performed on 2–4 fish, with 4 hybridisations (biological replicates) per group (16 hybridisations in total). Ellipses indicate 95% confidence intervals. The percentage of total variance explained by PC1 and PC2 is given in parentheses.

The principal component analysis (PCA) of the gill transcriptome profiles in rainbow trout from HH (high exposure/high response), HM (high exposure/moderate response), LL (low exposure/low response) and C (control, no exposure/no response) groups demonstrated a partial overlap between groups of fish exposed to *P. parvum* (HH, HM and LL) and their clear separation from the non-exposed control group (C) (**Figure 1**). This finding was reinforced by the differential gene expression analysis, which identified 1436, 1069 and 567 DEGs for comparisons of fish from HH vs control groups, HM vs control groups and LL vs control groups, respectively, with all DEGs referring to the human orthologs of fish genes (**Table 2**, **Supplementary Tables 1**–**3**, **Supplementary Figure 3**). The HH fish exposed to the high dose of *P. parvum* and displaying the strongest clinical response had the highest number of transcripts altered (1436 DEGs) followed by the similarly exposed but less affected HM fish (1069 DEGs), while the LL fish exposed to the low dose of *P. parvum* had approximately half of the number of transcripts altered (567 DEGs) relative to HH and HM groups.

**Figure 2:**
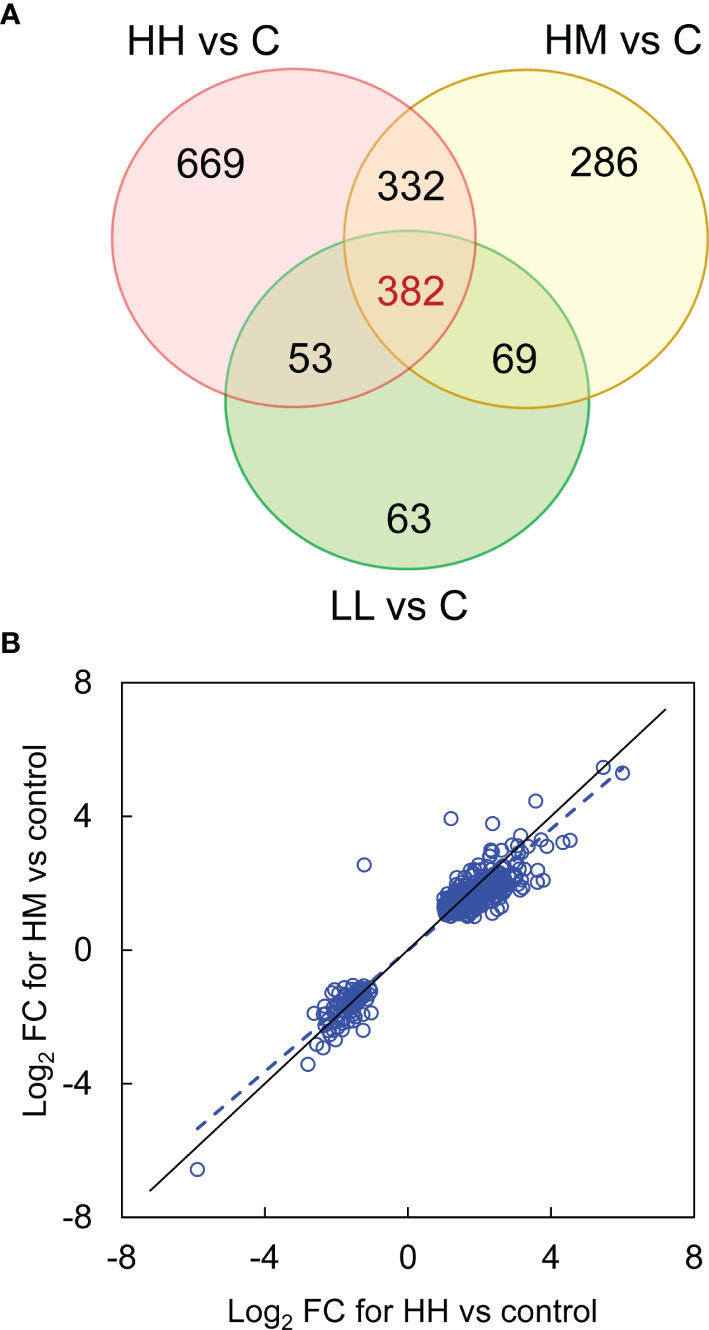
Differentially expressed genes (DEGs) in the gill transcriptome of rainbow trout from HH (high exposure/high response), HM (high exposure/moderate response) and LL (low exposure/low response) groups in relation to C (control, no exposure/no response) group. **(A)** Venn diagram showing the number of common (at intersections) and unique (outside intersections) DEGs for HH, HM and LL groups of fish. **(B)** Expression of 382 common DEGs in HM group plotted against HH group. The data were fitted with a reduced major axis line (blue dashed), shown along the line of equality (black solid). For details on common and unique DEGs see **Supplementary Table 4**.

Inspection of DEGs from the three groups of fish exposed to *P. parvum* (HH, HM and LL) revealed a relatively large number of common DEGs (382), despite two different doses of *P. parvum* used in the experiment (high and low) and three different phenotypic responses of fish to the toxic algae (high, moderate and low) (**Figure 2A**, **Supplementary Table 4**). The majority of the common genes (375 of 382) showed the same direction of change (288 genes upregulated and 87 genes downregulated) across the three groups of fish, with the magnitude of change (Log_2_ FC values) being highly correlated (correlation between HH and HM, r = 0.96, p < 0.001, **Figure 2B**; correlation between HH and LL, r = 0.91, p < 0.001, data not shown; correlation between HM and LL, r = 0.95, p < 0.001, data not shown). In addition, the mean expression of the 382 common genes was not significantly different between the groups, averaging 1.0 ± 1.7, 0.9 ± 1.6 and 0.8 ± 1.4 Log_2_ FC (mean ± standard deviation) for HH, HM and LL groups, respectively (one-way ANOVA, p = 0.090). The common genes with the highest levels of upregulation and downregulation are presented in **Table 3**.

**Table 3:** Top common genes altered in the gill transcriptome of rainbow trout from HH (high exposure/high response), HM (high exposure/moderate response) and LL (low exposure/low response) groups in relation to C (control, no exposure/no response) group.

Gene (HGNC symbol and name)	Gene expression (Log_2_ FC)		
	HH vs C	HM vs C	LL vs C
**CSF3**, colony stimulating factor 3	6.0	5.3	2.8
**GTPBP2**, GTP binding protein 2	5.5	5.5	5.9
**CXCL2**, C-X-C motif chemokine ligand 2	4.5	3.3	2.3
**ADAMTS1**, ADAM metallopeptidase with thrombospondin type 1 motif 1	4.3	3.2	2.0
**FOSL2**, FOS like 2, AP-1 transcription factor subunit	3.9	3.1	2.6
**MIOS**, meiosis regulator for oocyte development	−2.4	−2.9	−2.0
**ABCG2**, ATP binding cassette subfamily G member 2 (Junior blood group)	−2.6	−2.8	−1.6
**RAD21**, RAD21 cohesin complex component	−2.6	−1.9	−2.5
**DHRS4L2**, dehydrogenase/reductase 4 like 2	−2.8	−3.4	−2.4
**CCDC40**, coiled-coil domain containing 40	−5.9	−6.6	−6.9

Genes were considered differentially expressed at adjusted p-value < 0.05 and absolute Log_2_ FC > 1. For details on all 382 common DEGs see **Supplementary Table 4**.

### Canonical Pathways

**Figure 3:**
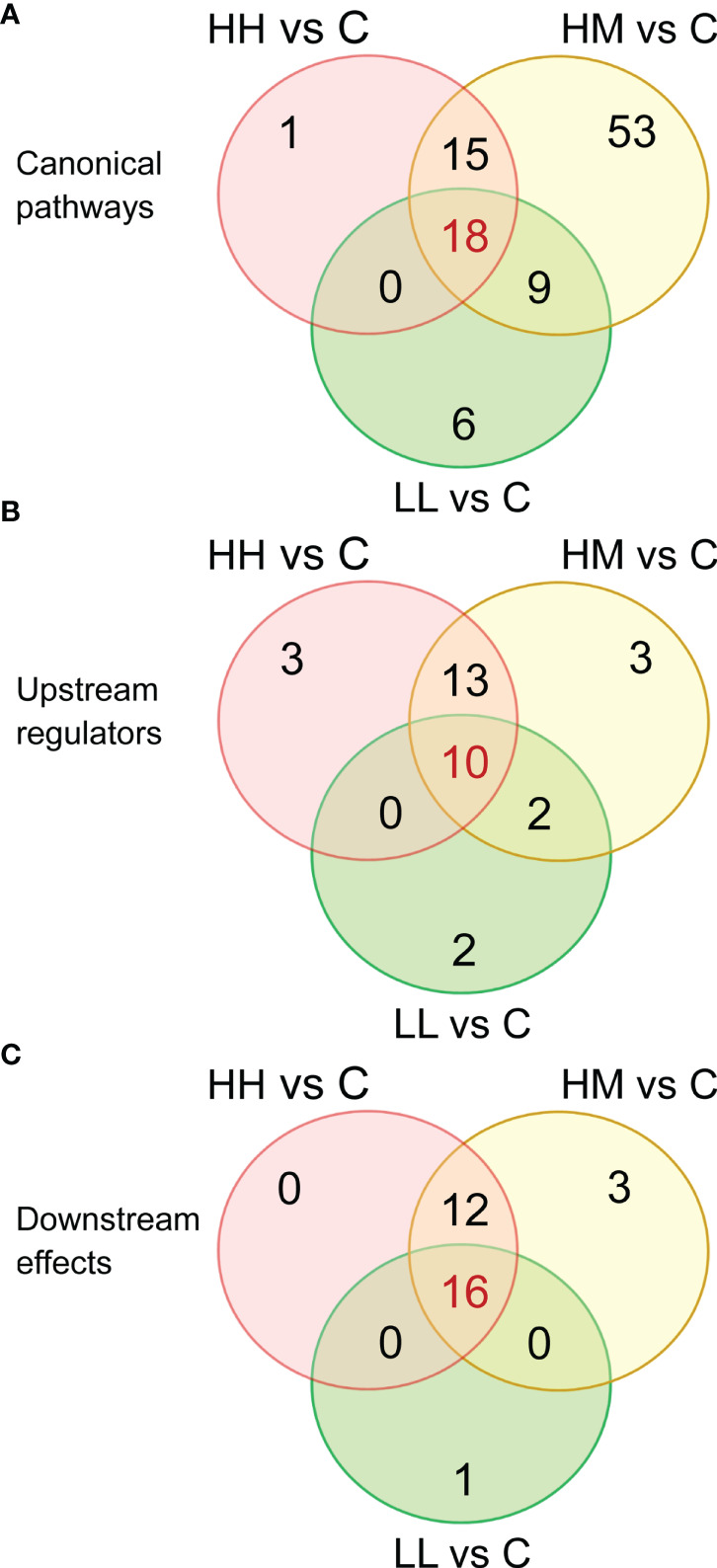
Venn diagrams showing the number of common and unique **(A)** canonical pathways, **(B)** upstream regulators and **(C)** downstream effects, identified by Ingenuity Pathway Analysis (IPA) in the gill transcriptome of rainbow trout from HH (high exposure/high response), HM (high exposure/moderate response) and LL (low exposure/low response) groups in relation to C (control, no exposure/no response) group. Functional analysis was performed on 1436, 1069 and 567 differentially expressed genes (DEGs) for comparisons HH vs C, HM vs C and LL vs C, respectively. For details on canonical pathways, upstream regulators and downstream effects see **Supplementary Tables 8**, **10** and **12**.

Based on the three sets of DEGs with 1436, 1069 and 567 transcripts, IPA identified 34, 95 and 33 canonical pathways that were significantly altered in the gill transcriptome of fish from HH, HM and LL groups, respectively, at B-H p-value < 0.001 (**Supplementary Tables 5**–**7**). Inspection of these pathways revealed a relatively large number of pathways (18) that were common between the three groups of fish (**Figure 3A**), consistent with the large number of common DEGs (**Figure 2A**).

**Figure 4:**
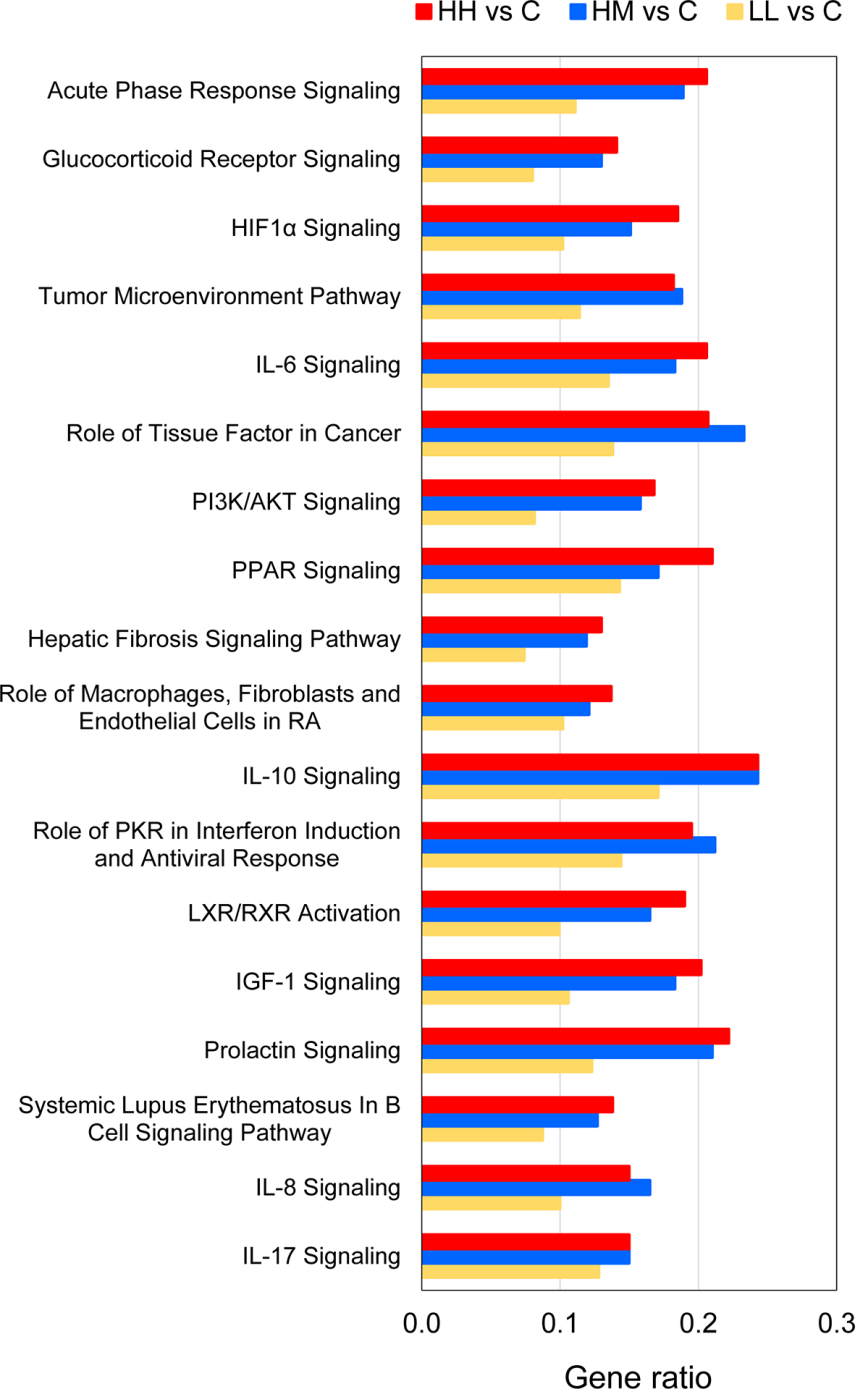
Common canonical pathways altered by *P. parvum* exposure in the gill transcriptome of rainbow trout from HH (high exposure/high response), HM (high exposure/moderate response) and LL (low exposure/low response) groups in relation to C (control, no exposure/no response) group. Pathways were identified as significantly altered by Ingenuity Pathway Analysis (IPA) at Benjamini-Hochberg multiple testing correction p-value < 0.001. Gene ratios refer to the number of DEGs contributing to each pathway (our experimental dataset) divided by the total number of genes that constitute the pathway (Ingenuity Knowledge Base). For details and list of corresponding genes see **Supplementary Tables 5**–**8**.

**Figure 5:**
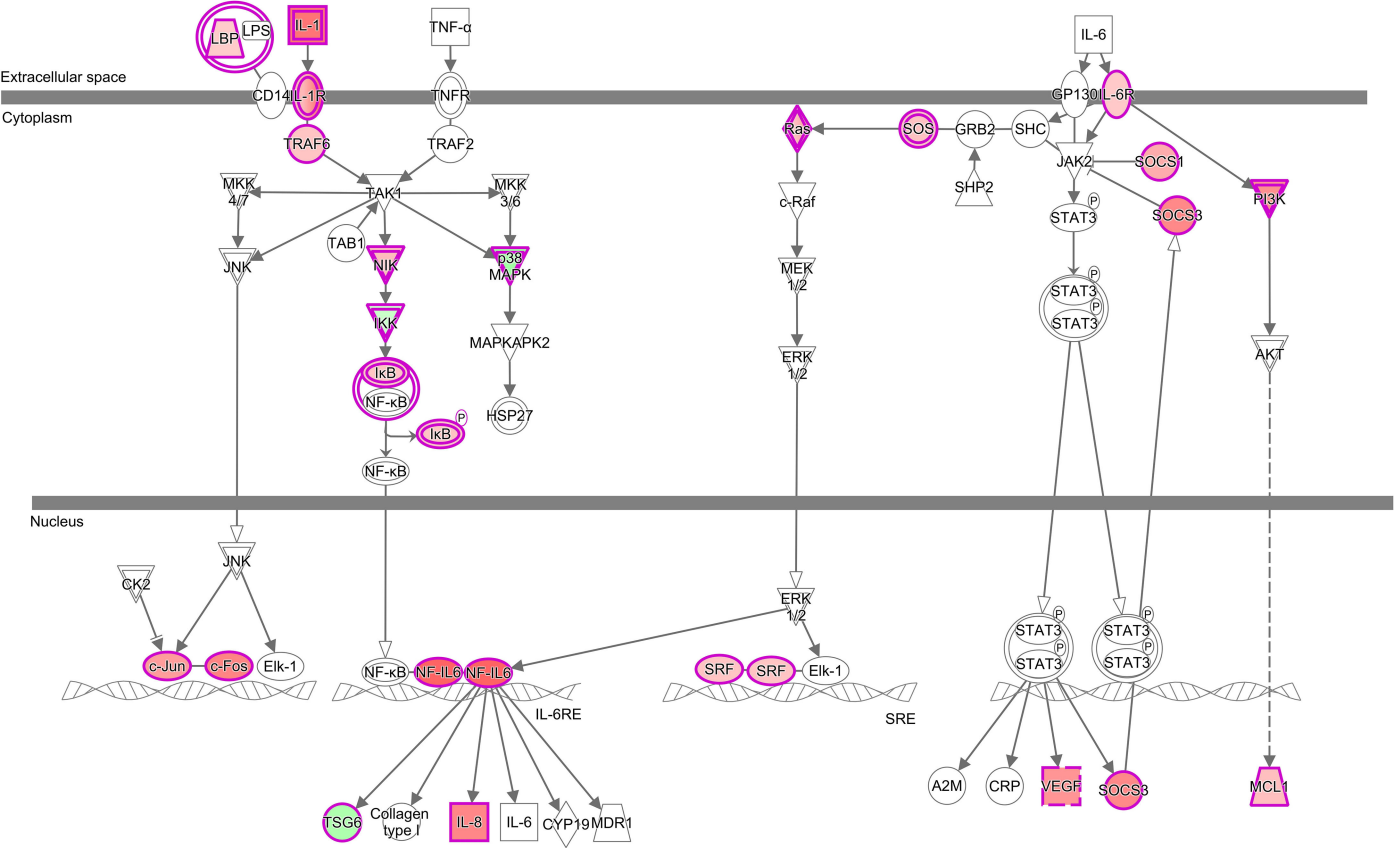
Alterations of IL-6 Signalling pathway in the gill transcriptome of rainbow trout exposed to the high dose of *P. parvum* with high phenotypic response (HH group) in relation to the non-exposed control fish (C group). The pathway was identified as significant (Benjamini-Hochberg multiple testing correction p-value < 0.001) and activated (z-score ≥ 2) by Ingenuity Pathway Analysis (IPA). Among 126 genes that constitute the pathway, 26 were significantly altered by *P. parvum* exposure (yielding the gene ratio of 0.206), including 23 genes upregulated (in red) and 3 genes downregulated (in green). The upregulated genes were CEBPB, CCAAT enhancer binding protein beta; FOS, Fos proto-oncogene; AP-1 transcription factor subunit; HRAS, HRas proto-oncogene; GTPase; IL1B, interleukin 1 beta; IL1R2, interleukin 1 receptor type 2; IL1RAPL1, interleukin 1 receptor accessory protein like 1; IL1RAPL2, interleukin 1 receptor accessory protein like 2; IL6R, interleukin 6 receptor; IL8/CXCL8, C-X-C motif chemokine ligand 8; JUN, Jun proto-oncogene; AP-1 transcription factor subunit; LBP, lipopolysaccharide binding protein; MAP4K4, mitogen-activated protein kinase kinase kinase kinase 4; MCL1, MCL1 apoptosis regulator; BCL2 family member; NFKBIA, NFKB inhibitor alpha; PIK3R5, phosphoinositide-3-kinase regulatory subunit 5; RAP2B, RAP2B; member of RAS oncogene family; RASD1, ras related dexamethasone induced 1; SOCS1, suppressor of cytokine signalling 1; SOCS3, suppressor of cytokine signalling 3; SOS1, SOS RasRac guanine nucleotide exchange factor 1; SRF, serum response factor; TRAF6, TNF receptor associated factor 6 and VEGFA, vascular endothelial growth factor A. The downregulated genes were IKBKAP/ELP1, elongator acetyltransferase complex subunit 1; MAPK13, mitogen-activated protein kinase 13 and TNFAIP6, TNF alpha induced protein 6. For details see **Supplementary Tables 1**, **5** and **8**.

The 18 common pathways were predominantly associated with the cellular immune response and cytokine signalling, as evidenced by significant alterations of Acute Phase Response Signalling, IL-6 Signalling, IL-10 Signalling, Role of PKR in Interferon Induction and Antiviral Response, IL-8 Signalling and IL-17 Signalling (**Figure 4**, **Supplementary Table 8**). Among them, IL-6 Signalling (**Figure 5**) and IL-17 Signalling (**Supplementary Figure 4**) were both significantly activated, based on their IPA z-score > 2 for fish from HH, HM and LL groups. According to the predictions made by IPA, binding of cytokines to their specific receptors induced significant changes in intracellular and second messenger signalling pathways (represented by Glucocorticoid Receptor Signalling) and nuclear receptor signalling pathways (represented by PPAR Signalling and LXR/RXR Activation). The gills of fish exposed to *P. parvum* were likely affected by hypoxia, as indicated by nearly fully activated (z-scores from 0.9 to 1.9) HIF1α Signalling pathway, which is consistent with the increased production of gill mucus observed in all exposed fish (**Table 1**, **Supplementary Figure 5**) and the altered expression of a number of mucin transcripts, including MUC16 and MUC21 (upregulated) and MUC2, MUC5 and MUC7 (downregulated) (**Supplementary Table 4**). The *P. parvum*-induced cellular stress and injury were also supported by alterations of IGF-1 Signalling and Prolactin Signalling pathways, both promoting cellular proliferation and differentiation of a variety of cell types in the attempts to repair the damaged tissue. Finally, 3 of the 18 common pathways were related to human inflammatory diseases (Role of Macrophages, Fibroblasts and Endothelial Cells in Rheumatoid Arthritis, Systemic Lupus Erythematosus in B Cell Signalling Pathway and Hepatic Fibrosis Signalling Pathway) and 3 others were associated with cancer (Tumour Microenvironment Pathway, Role of Tissue Factor in Cancer and PI3K/AKT Signalling).

Comparison of the gene ratios (the number of DEGs contributing to the pathway divided by the total number of genes that constitute the pathway) for the 18 common pathways across the three groups of fish demonstrated that the fish exposed to the high dose of *P. parvum* (HH and HM) had consistently similar ratios, and thus a similar number of DEGs contributing to a given pathway, while the gene ratios observed in the fish exposed to the low dose of the toxic algae were substantially lower (**Figure 4**, **Supplementary Table 8**).

### Upstream Regulators

IPA upstream regulator analysis identified 26, 28 and 14 upstream regulators (excluding chemicals and drugs) that could explain changes in the gene expression patterns observed in the gill tissue of HH, HM and LL fish, respectively, following the exposure to *P. parvum* (**Supplementary Table 9**). These upstream regulators were considered significant at Benjamini-Hochberg multiple testing correction p-value < 1E−15 and absolute z-score ≥ 2. Among the identified upstream regulators, 10 of them were shared between the three groups of fish (**Figure 3B**).

**Figure 6:**
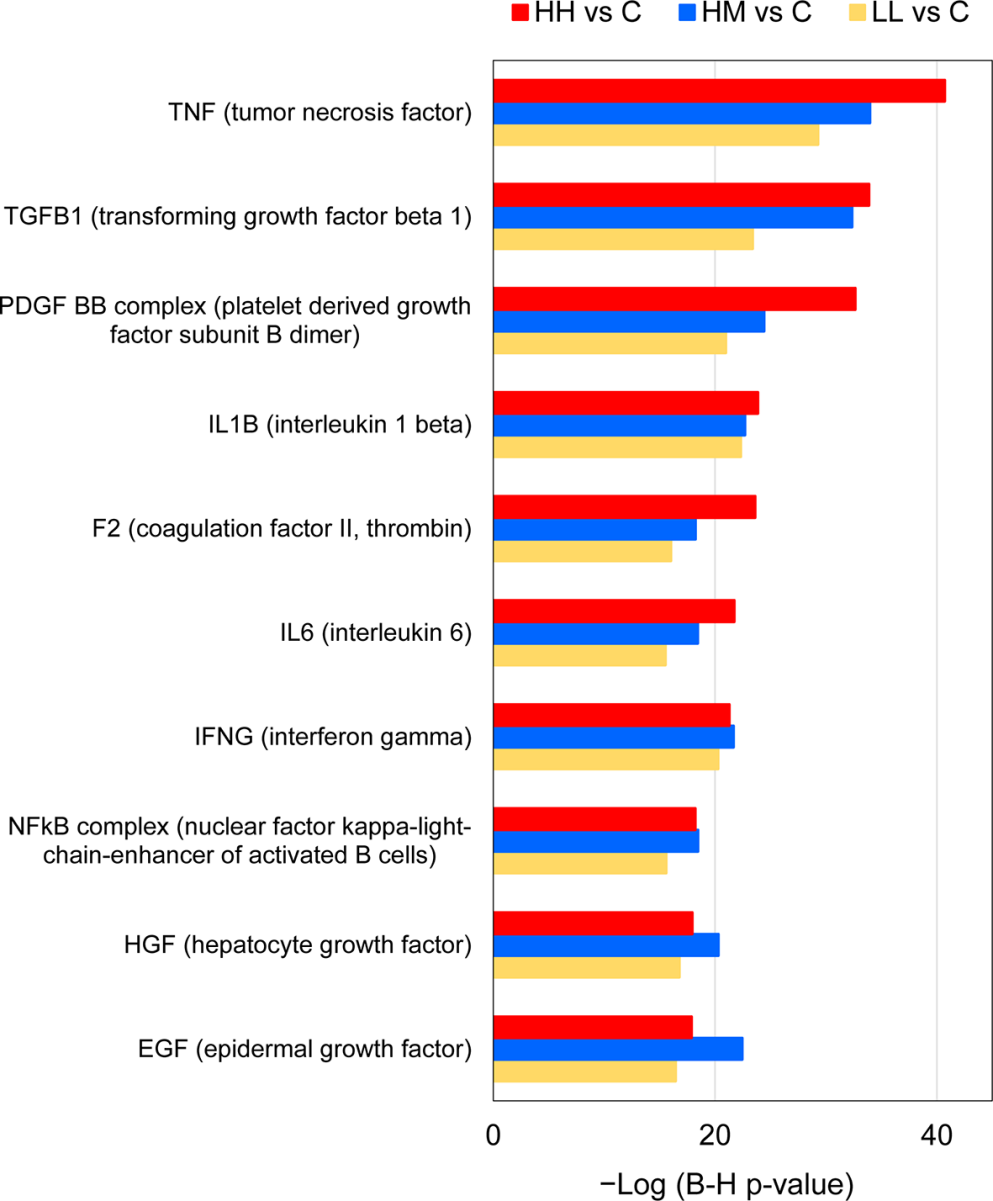
Common upstream regulators of gene expression changes in the gills of rainbow trout exposed to *P. parvum* from HH (high exposure/high response), HM (high exposure/moderate response) and LL (low exposure/low response) groups in relation to C (control, no exposure/no response) group, predicted by Ingenuity Pathway Analysis (IPA). Upstream regulators were considered significant at Benjamini-Hochberg multiple testing correction p-value < 1E−15 and absolute z-score ≥ 2, excluding chemicals and drugs (for details see **Supplementary Tables 9** and **10**).

The 10 common upstream regulators (z-scores from 2.7 to 5.5) included 4 cytokines (TNF, IL-1B, IL-6 and IFNG), with TNF predicted to be responsible for expression of 284, 223 and 143 target genes (DEGs) in the gill transcriptome of HH, HM and LL fish, respectively (**Figure 6**, **Supplementary Table 10**). Among the common upstream regulators were also 3 growth factors (TGFB1, HGF and EGF), 2 complexes (PDGF BB and NFkB) and a peptidase (F2). One common upstream regulator (IL-6) was consistently upregulated across the three groups of fish (Log_2_ FC values from 2.1 to 2.7), while others were not significantly altered in our experimental dataset (apart from F2 transcript downregulated in HM fish).

### Downstream Effects

IPA downstream effect analysis predicted 28, 31 and 17 downstream effects based on the gene expression changes observed in the gill tissue of HH, HM and LL fish, respectively, following the exposure to *P. parvum* (**Supplementary Table 11**). The downstream effects were considered significant at Benjamini-Hochberg multiple testing correction p-value < 1E−15. A relatively large number of downstream effects (16) were common between the three groups of fish (**Figure 3C**).

**Figure 7:**
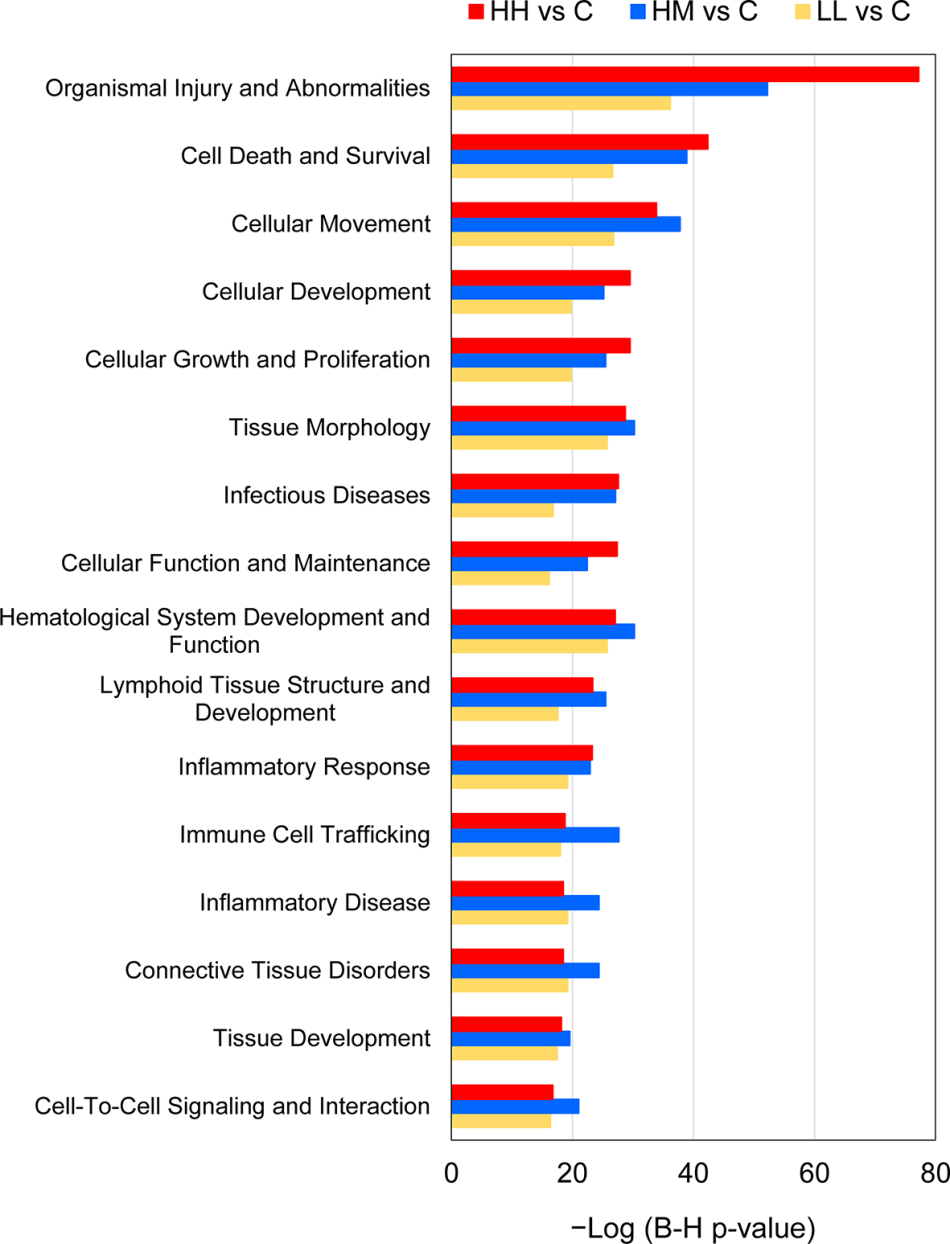
Common downstream effects of gene expression changes in the gills of rainbow trout exposed to *P. parvum* from HH (high exposure/high response), HM (high exposure/moderate response) and LL (low exposure/low response) groups in relation to C (control, no exposure/no response) group, predicted by Ingenuity Pathway Analysis (IPA). Downstream effects were considered significant at Benjamini-Hochberg multiple testing correction p-value < 1E−15 (for details see **Supplementary Tables 11** and **12**).

The predicted 16 common downstream effects covered cellular (7 effects), tissue and organ (5 effects) and organismal (4 effects) levels of organisation (**Figure 7**, **Supplementary Table 12**). At the cellular level, the prediction included changes in Cell Death and Survival, Cellular Movement, Cellular Development, Cellular Growth and Proliferation, Cellular Function and Maintenance, Cell-To-Cell Signalling and Interaction, and Immune Cell Trafficking. At the tissue and organ levels, the prediction included alterations of Tissue Morphology, Haematological System Development and Function, Lymphoid Tissue Structure and Development, Connective Tissue Disorders, and Tissue Development. Finally, the gene expression changes in HH, HM and LL fish pointed towards the whole-animal downstream effects such as Organismal Injury and Abnormalities, Infectious Diseases, Inflammatory Response, and Inflammatory Disease. Among all 16 common downstream effects, Organismal Injury and Abnormalities was characterized by the highest number of contributing genes, i.e., 1403, 1043 and 557 from HH, HM and LL fish, respectively.

## Discussion

Climate change is altering aquatic ecosystems worldwide, including patterns, distribution, and intensity of HABs in marine, brackish, and freshwater environments (19, 20). These effects have been linked to 1) HAB species becoming more competitive relative to non-HAB species within plankton communities, 2) increased toxin production by toxic HAB species, and 3) HAB species reaching higher biomass due to changes in hydrology (55). Blooms present a challenge to fish health particularly in aquaculture, where containment of fish prevents avoidance behaviours. In anticipation of growing HAB problems, intensified research is needed to uncover molecular mechanisms by which toxin-producing algae affect wild and farmed fish, with the use of controlled laboratory settings, cultured HAB species and high-throughput technologies. Furthermore, there is also a need to perform some experiments at the level of whole animals. Although valuable information can be gained from experiments on fish cell lines, they lack physiological milieu and behavioural responses that are important in toxicological studies (56). We are the first to report on the gill transcriptomic responses to high and low sublethal doses of the toxin-producing *P. parvum* in commercially important rainbow trout, taking into account the fish phenotype.

Visual inspection of the fish following exposure to *P. parvum* allowed for identification of two distinct phenotypes among the fish originating from the same tanks and exposed to the high dose of the toxin-producing alga (**Table 1**). Fish with a more advanced phenotype (HH) showed advanced lethargy, loss of balance and dark skin colour, while fish with a less advanced phenotype (HM) showed only mild lethargy, with both phenotypes having increased respiratory effort and increased production of gill mucus. Our interpretation of these differences is that fish with the more advanced phenotype were likely metabolically more active than fish with the less advanced phenotype. Fish with higher metabolic rate and thus higher oxygen uptake have been demonstrated to 1) increase water flow over the gill by adjusting the volume and frequency of buccal pumping, 2) increase blood flow inside the gill to alter the perfusion levels of lamellae, and 3) initiating remodelling of gill tissue towards more protruded lamellae (16, 57). All these adjustments make the gill tissue potentially more available for toxin uptake (58). Salmonids are particularly known for their substantial intraspecific variation in metabolic rate, which has been linked to the differences in individual’s behaviour (e.g., dominance, foraging or stress avoidance) and performance (e.g., growth) (59–61). With our single sampling approach at 4–5 h post-exposure, we were unable to establish whether the phenotypic differences persisted or converged with time. In contrast to the high dose exposure, no phenotypic differences were observed among the fish exposed to the low dose of *P. parvum* (**Table 1**). Similarly, we cannot exclude the possibility that different metabolic phenotypes might manifest themselves at the lower dose over a more prolonged period of time.

Despite the differences in the phenotype, fish exposed to the high dose of *P. parvum* (HH and HM) had a relatively similar number of genes altered (1436 and 1069) in the gill tissue, while the fish exposed to approximately half of the dose of *P. parvum* (LL) had approximately half of the genes altered (567) relative to HH and HM groups (**Figure 2A**, **Table 2**). The positive relationship between the dose of *P. parvum* and the number of DEGs points towards an overall sensitivity of the gill transcriptome to toxic algae dose. Dose-dependent transcriptomic effects have also been demonstrated in the gut of Atlantic salmon exposed to plant proteins (49) and recently in human nasal airway epithelium cultures exposed to air pollution particulate matter < 2.5 μm (PM_2.5_) (62). Furthermore, fish from HH and HM groups had not only twice more DEGs identified in their gills relative to LL fish, but also twice as many upstream regulators (26, 28 and 14, respectively) (**Figure 3B**) and twice as many downstream effects (28, 31 and 17, respectively) (**Figure 3C**). In contrast, fish exposed to the high dose of *P. parvum* differed in the number of canonical pathways, with only 34 pathways identified in the gill of fish showing the more advanced phenotype (HH) and 95 pathways identified in the gill of fish with the less advanced phenotype (HM) (**Figure 3A**). Fewer DEGs contributing to more pathways (1069 and 95, respectively) may reflect more coordinated patterns of gene expression in HM fish, while more DEGs contributing to fewer pathways (1436 and 34, respectively) may suggest the onset of dysregulated gene expression patterns in HH fish. The transition from coordinated to dysregulated gene expression patterns has been linked to tissue and organ malfunction, leading to disease and ultimately death (63, 64). Thus, the high dose of *P. parvum* used in our experiment may be considered as the borderline of what the fish could handle, with the HH phenotype representing individuals that were pushed beyond their physiological control and the HM phenotype reflecting fish that were still able to cope with the algal exposure by mobilizing a broader range of defence mechanisms initiated at the level of gill transcriptome. The fact that the LL phenotype shared many of the altered canonical pathways as well as upstream regulators and downstream effects with the HH and HM fish may reflect a more general and balanced response of the LL fish to the *P. parvum* exposure. The mucus production and release from gills of all exposed fish likely represent a physical defence mechanism for clearing the gills from algae/algal toxin. Accordingly, few algae were found in direct contact with the gills while high numbers of the motile algal cells were found trapped in the secreted mucus (**Supplementary Figure 5**).

The gill transcriptomic responses to two different doses of *P. parvum* in fish expressing three different phenotypes were to a relatively large extent overlapping. The total number of unique DEGs for HH, HM and LL fish was 1854, with 382 (20.6%) of them being common (**Figure 2A**) and having highly correlated gene expression patterns (**Figure 2B**). Likewise, the three groups of fish shared 18 (17.6%) of 102 canonical pathways (**Figure 3A**), 10 (30.3%) of 33 upstream regulators (**Figure 3B**) and 16 (50.0%) of 32 downstream effects (**Figure 3C**), according to the functional analysis of gene expression performed by IPA. These overlaps are important to investigate because they likely represent a core transcriptomic response characterizing the gill’s attempt to maintain homeostasis when responding to *P. parvum*, regardless of the algal dose and fish phenotype. A similar approach has been recently used to gain insights into the mechanisms of multifactorial gill disease in Atlantic salmon (53), adaptation of the foodborne pathogen *Listeria monocytogenes* to desiccation (65) and resilience to heat stress in laying hens (66).

DEGs identified as common for HH, HM and LL fish (382 transcripts in total) represent a diverse group of genes, encoding 23 cell surface receptors (including 7 G-protein coupled receptors), 30 transporters, 2 ion channels, 11 cytokines (including CSF3 and CXCL2 with the highest Log_2_ FC values of all DEGs), 128 enzymes (including 17 peptidases, 13 kinases and 10 phosphatases), 3 growth factors, 54 transcription factors (including 2 ligand-dependent nuclear receptors) and 4 translation regulators among others (**Table 3**, **Supplementary Table 4**). Such diversity is consistent with the cellular and molecular mechanisms of action by which most toxins disrupt eukaryotic cells, first interacting with the host cell surface and then compromising the host intracellular processes associated with energy metabolism, cytoskeleton stability, gene expression, posttranslational modifications, motility, secretion, cell division or other more specific functions (67, 68). Some toxins are known to specifically disrupt the ionic equilibrium maintained by the cell membrane barrier, which can be done either directly (by forming pores or causing an enzymatic degradation of the lipid bilayer) or indirectly – by acting on ion pumps or ion-gated channels that are responsible for maintaining the ion gradients (69). A classic example of the pore-forming toxins is pardaxin secreted by the finless sole (*Pardachirus marmoratus*), which is used as a defensive mechanism against predators including sharks *via* targeting their gills (70). Other toxins interfere with the signalling cascades of the eukaryotic cells by acting on a variety of cell membrane targets, including ligand-gated ionotropic receptors, G-protein coupled receptors, tyrosine kinase receptors, integrin receptors and certain lipid species present in the bilayer plasma membrane of the cell. Examples here are domoic acid produced by the red alga *Chondria armata* that acts on ligand-gated ionotropic glutamate receptors (71) and okadaic acid produced by the marine algae *Halichondria okadai* and *Halichondria melandocia* that affects protein kinases and phosphatases (69). In our study, some of the DEGs identified as common for HH, HM and LL fish (e.g., ion channel and transporter transcripts) clearly point towards *P. parvum* disrupting the ionic regulation of the gill tissue, while other common DEGs (e.g., kinase and phosphatase transcripts) implicate the dysregulation of the signalling cascades in the gill cells. The wide range of gill transcriptomic responses to *P. parvum* likely reflect the heterogeneity of effects of the algal cells themselves and their released toxic compounds along with counteractive reactions in the gills such as mucus production and secretion, tissue repair and innate immune reactions.

Inspection of 18 canonical pathways identified as commonly altered in HH, HM and LL fish suggests that exposure to *P. parvum* promotes a strong inflammatory response within the gill tissue and thus induces gill inflammation, as evidenced by Acute Phase Response Signalling, IL-6 Signalling, IL-8 Signalling and IL-17 Signalling pathways (**Figure 4**, **Supplementary Table 8**). Whether these changes were associated with the resident immune cells (GIALT) or the recruitment of new immune cells to the gill tissue remains unknown. The acute phase response is a rapid inflammatory reaction that provides protection against microorganisms by non-specific defence mechanisms (72). It is typically manifested by an increase in inflammatory factors (such as pro-inflammatory cytokines) and a change in concentration of several plasma proteins (the acute phase proteins) that can become measurable as early as 4-5 h after a single inflammatory stimulus. One of the key regulators of the acute phase response is IL-6 (a pleiotropic cytokine with roles in inflammation, immune response, haematopoiesis, and the endocrine and nervous systems), which signals through JAK-STAT and RAS-MAPK pathways to regulate transcription of target genes (73, 74). In our study, 26 (HH), 23 (HM) and 17 (LL) of the 126 genes that constitute the IL-6 Signalling pathway were significantly altered, with the overall direction of change pointing towards a significant activation of this pathway in the three groups of fish (**Figure 5**, **Supplementary Tables 5**–**8**). Among the target genes of IL-6 is IL-8, a member of the C-X-C family of chemokines that plays a central role in inflammation, angiogenesis, and tumour growth, with the IL-8 receptors expressed on several cell types like neutrophils, monocytes, endothelial cells, and tumour cells (75). Signalling by IL-8 or other ligands of the IL-8 receptors can trigger inflammation in cells like neutrophils leading to chemotaxis, the respiratory burst, granule release, and increased cell adhesion (76). All fish exposed to *P. parvum* showed a robust upregulation of IL-8 transcription (with Log_2_ FC values from 1.3 to 2.3), along with highly significant alteration of IL-8 Signalling consistent with activation (z-scores from 1.8 to 3.4) (**Supplementary Tables 4** and **8**). Another highly versatile cytokine with a strong pro-inflammatory profile is IL-17, which promotes expansion and recruitment of innate immune cells such as neutrophils and stimulates production of beta-defensins and other anti-microbial peptides (77). Because IL-17 is primarily secreted by T cells, it plays a central role in integrating adaptive and innate immune responses (78). The IL-17 Signalling pathway was fully activated in HH, HM and LL fish (z-scores > 3.0), with 28 (HH), 28 (HM) and 24 (LL) of the 187 genes that make up the pathway being significantly altered, including CSF3 with the highest Log_2_ FC values of all 382 common DEGs (**Table 3**, **Supplementary Figure 4**). Although the interpretation of our results by IPA in the context of human inflammatory diseases may be irrelevant, the presence of highly affected pathways related to autoimmune diseases (i.e., Role of Macrophages, Fibroblasts and Endothelial Cells in Rheumatoid Arthritis, Systemic Lupus Erythematosus in B Cell Signalling Pathway and Hepatic Fibrosis Signalling Pathway) highlight the severity and scope of the gill inflammatory response towards *P. parvum* (**Figure 4**). A similar argument can be made for the three cancer-related pathways (i.e., Tumour Microenvironment Pathway, Role of Tissue Factor in Cancer and PI3K/AKT Signalling). The exposure to *P. parvum* does not induce cancer, but some of the key features associated with cancer (such as local inflammation, cell proliferation, angiogenesis, and hypoxia) are also the functions of the DEGs identified in the current study (79).

If not resolved, the acute inflammatory response may contribute to tissue injury and chronic inflammation, an underlying cause of human chronic inflammatory diseases such as arthritis, diabetes, metabolic syndrome, neurodegenerative diseases, asthma, allergy, tissue fibrosis, certain types of cancer, cardiovascular and periodontal diseases (80). Resolution of inflammation involves highly coordinated actions of various immune and non-immune cells and pathways to first clear damaged cells and pro-inflammatory cytokines (by apoptosis and efferocytosis) and then at least partially reconstitute tissue integrity and function (81). In our study, the attempts to resolve inflammation in the gill tissue following exposure to *P. parvum* are evidenced by Glucocorticoid Receptor Signalling and IL-10 Signalling pathways, commonly altered in HH, HM and LL fish (**Figure 5**, **Supplementary Tables 5**–**8**). Glucocorticoids are a major subclass of steroid hormones that produce their effects on responsive cells by activating the glucocorticoid receptor to modulate the transcription of target genes, thereby regulating a large number of immune, metabolic, cardiovascular and behavioural functions (82). Despite the ability of glucocorticoids to induce transcription of anti-inflammatory genes, the main anti-inflammatory effects of glucocorticoids are through repression of pro-inflammatory cytokine genes (83). These anti-inflammatory actions are also complemented by the ability of glucocorticoids to induce apoptosis of immune cells, including monocytes and T cells. Among the 462 genes that constitute the Glucocorticoid Receptor Signalling pathway, 65, 60 and 37 genes were altered in HH, HM and LL fish, respectively, including IL-10 transcription with Log_2_ FC values from 2.3 to 2.5 (**Supplementary Table 4**). IL-10 is a cytokine with diverse effects on hematopoietic cells, which regulates the growth and differentiation of B, T, natural killer and dendritic cells (84). One of its best-known functions is to limit and terminate the inflammatory response. The mechanism behind this action is signalling through the IL-10 receptor that causes inhibition of JAK/STAT-dependent signalling, leading to the transcription inhibition of pro-inflammatory genes like IL-1 and TNF-α (85). In our study, both pro- and anti-inflammatory cytokine transcripts (**Supplementary Table 4**) and signalling pathways (**Supplementary Table 8**) were altered, but because we used a single sampling approach (with no temporal resolution of gene expression data), it is impossible to conclude whether the inflammation of gill tissue following the 4–5 h exposure to *P. parvum* was progressing or resolving.

The presence of gill inflammation inferred from the gene expression changes in the fish exposed to *P. parvum* is also supported by the results of the IPA upstream regulator analysis (**Figure 6**, **Supplementary Table 10**). Specifically, 6 of the 10 common upstream regulators (all identified as activated) are known for their pro-inflammatory action, including TNF, TGFB1, IL-1B, IL-6, IFNG and NFkB complex, with TGFB1 and NFkB complex also contributing to mounting the anti-inflammatory response (73, 86–89). The remaining 4 common upstream regulators have been linked to inhibition of inflammatory responses (PDGF BB), inflammation-induced coagulation (F2) and the growth, proliferation and differentiation of numerous cell types that are necessary for tissue repair and regeneration (HGF and EGF) (90–92). It is important to clarify here that the IPA upstream analysis does not take into account the gene expression observed for the predicted upstream regulator itself, because the gene expression for the upstream regulator may not differ between the experimental and control groups. In our study, only one upstream regulator transcript (IL-6) was commonly upregulated in the fish exposed to *P. parvum*, which suggests that the remaining 9 common upstream regulators were likely activated by other means rather than by increased gene expression.

Finally, the results of the IPA downstream effect analysis are consistent with gill inflammation inferred from the transcriptomic data, as evidenced by the common whole-animal downstream effects such as Inflammatory Response, Inflammatory Disease, and Infectious Diseases (**Figure 7**, **Supplementary Table 12**). These predictions are in good agreement with the secretion of gill mucus observed in all fish exposed to *P. parvum* (**Table 1**, **Supplementary Figure 5**). Furthermore, gill inflammation in salmonids is characterised by excessive cellular death by apoptosis, followed by proliferation and migration of epithelial cells to replace the damaged mucosal surface and to provide a defensive barrier that isolates the gill tissue from the environmental insults (93, 94). These processes were in our study represented by a number of predicted cellular downstream effects that were common for all fish exposed to *P. parvum*, including Cell Death and Survival, Cellular Movement, Cellular Development, Cellular Growth and Proliferation, Cellular Function and Maintenance, Cell-To-Cell Signalling and Interaction, and Immune Cell Trafficking. The cellular effects pointing towards the inflammatory processes were further supported by the tissue and organ downstream effects such as Tissue Morphology, Haematological System Development and Function, Lymphoid Tissue Structure and Development, Connective Tissue Disorders, and Tissue Development, providing a strong support for harmful algal gill pathology in rainbow trout exposed to *P. parvum* (95, 96).

Our results have important implications for understanding the molecular basis of HAB-induced gill injury, which has remained largely unknown due to the lack of high-throughput transcriptomic studies performed on teleost fish under controlled laboratory conditions. Firstly, we demonstrated that the gill transcriptomic responses are sensitive and proportional to the sublethal concentrations of *P. parvum*. However, the whole-animal phenotypic and transcriptomic responses to near-lethal algal levels may vary, depending on whether the individual fish are able to maintain physiological homeostasis as observed for HH vs HM phenotypes. Thus, the phenotype variabilities for a given algal exposure should be taken into account when evaluating the effects of sublethal doses of HABs on fish. Secondly, we identified more coordinated patterns of gill gene expression in fish with relatively healthy phenotypes (LL and HM fish), and less coordinated (more dysregulated) patterns of gill gene expression in HH fish with advanced clinical presentation. The distinction between the coordinated and dysregulated gene expression patterns was based on the number of DEGs in relation to the number of canonical pathways identified as significantly altered, highlighting the importance of functional analysis of gene expression in aquatic toxicology studies. Thirdly, we demonstrated that the transcriptomic changes in the gill tissue of fish exposed to different doses of *P. parvum* (high and low) and expressing three different phenotypes (high, moderate and low responses) showed a high degree of overlap, pointing towards common molecular defence mechanisms against *P. parvum*. Finally, the inspection of the common transcriptomic features (i.e., DEGs, canonical pathways, upstream regulators, and downstream effects) in the gills of HH, HM and LL fish led us to the conclusion that *P. parvum* caused a strong inflammatory response, associated with gill inflammation and the attempts to calm the inflammation down, as well as potential downstream tissue damage and defects in gill function. While we could not determine if the inferred gill inflammation was progressing or resolving, our study clearly suggests that even HABs with the sublethal levels of toxicity may contribute to the serious gill disorders in rainbow trout. By providing insights into the gill transcriptomic responses to toxin-producing *P. parvum* in teleost fish, our research opens new avenues for investigating how to monitor and mitigate toxicity of HABs before they become lethal.

## Data Availability Statement

The datasets generated for this study are available in the ArrayExpress repository (http://www.ebi.ac.uk/arrayexpress/) under accession numbers A-MEXP-2315 (Trout_imm_v1 array design) and E-MTAB-10541 (microarray raw data).

## Ethics Statement

All animal procedures were in agreement with the EU Directive 2010/63/EU for animal experiments and performed with permission from the Danish Animal Experiments Inspectorate, license 2016-15-0201-00864. The experiment was approved by the School of Biology Ethics Committee at the University of St Andrews (reference SEC21041).

## Author Contributions

NL, PH, NA, and SM conceived and designed the study, with the input from AB and DF. DS and NL performed the animal work. SM oversaw molecular work, done by MC and EK. MC carried out differential expression analysis in R. EK performed functional analysis of gene expression and deposited raw microarray data at ArrayExpress. EK, MC, and SM drafted and prepared the manuscript for publication. All authors contributed to the article and approved the submitted version.

## Funding

This work was supported by the BBSRC EastBio PhD studentship awarded to MC, the Danish Strategic Research Council grant No 060300449B HABFISH, and the European Maritime and Fisheries Fund and the Danish Fisheries Agency joint grant “Sundt Dambrug”. Molecular work at University of Aberdeen was funded by Scottish Aquaculture Innovation grant SL 2017 08. EK was supported by BBSRC grant BB/R018812/1.

## Conflict of Interest

The authors declare that the research was conducted in the absence of any commercial or financial relationships that could be construed as a potential conflict of interest.

## Publisher’s Note

All claims expressed in this article are solely those of the authors and do not necessarily represent those of their affiliated organizations, or those of the publisher, the editors and the reviewers. Any product that may be evaluated in this article, or claim that may be made by its manufacturer, is not guaranteed or endorsed by the publisher.

## Supplementary Material

Supplementary Figure 1 3D PCA (principal component analysis) plot of the gill transcriptome profiles in rainbow trout from HH (high exposure/high response), HM (high exposure/moderate response), LL (low exposure/low response) and C (control, no exposure/no response) groups. Each circle refers to all genes from one microarray hybridisation performed on 2–4 fish, with 4 hybridisations (biological replicates) per group (16 hybridisations in total). The percentage of total variance explained by PC1, PC2 and PC3 is given in parentheses.

Supplementary Figure 2 Principal component analysis (PCA) of the gill transcriptome profiles in rainbow trout exposed to *P. parvum* (HH, HM and LL groups) and non-exposed control fish. Each circle refers to all genes from one microarray hybridisation performed on 2–4 fish, with 12 hybridisations representing the exposed fish and 4 hybridisations representing non-exposed fish. Ellipses indicate 95% confidence intervals. The percentage of total variance explained by PC1 and PC2 is given in parentheses.

Supplementary Figure 3 Volcano plots of differential expression of RNA targets in the gill transcriptome of rainbow trout from HH (high exposure/high response), HM (high exposure/moderate response) and LL (low exposure/low response) groups in relation to C (control, no exposure/no response) group. Genes were considered differentially expressed at adjusted p-value < 0.05 and absolute Log_2_ FC > 1. Upregulated genes are in green, while downregulated genes are in red.

Supplementary Figure 4 Alterations of IL-17 Signalling pathway in the gill transcriptome of rainbow trout exposed to the high dose of *P. parvum* with high phenotypic response (HH group) in relation to the non-exposed control fish (C group). The pathway was identified as significant (Benjamini-Hochberg multiple testing correction p-value < 0.001) and activated (z-score ≥ 2) by Ingenuity Pathway Analysis (IPA). Among 187 genes that constitute the pathway, 28 were significantly altered by *P. parvum* exposure (yielding the gene ratio of 0.150), including 23 genes upregulated (in red) and 5 genes downregulated (in green). The upregulated genes were CEBPB (CCAAT enhancer binding protein beta), CSF3 (colony stimulating factor 3), FOS (Fos proto-oncogene, AP-1 transcription factor subunit), HRAS (HRas proto-oncogene, GTPase), HSP90AA1 (heat shock protein 90 alpha family class A member 1), HSP90AB1 (heat shock protein 90 alpha family class B member 1), IL11 (interleukin 11), IL12B (interleukin 12B), IL1B (interleukin 1 beta), IL8/CXCL8 (C-X-C motif chemokine ligand 8), JUN (Jun proto-oncogene, AP-1 transcription factor subunit), LTB (lymphotoxin beta), MMP13 (matrix metallopeptidase 13), MMP3 (matrix metallopeptidase 3), MMP9 (matrix metallopeptidase 9), NOS2 (nitric oxide synthase 2), PIK3R5 (phosphoinositide-3-kinase regulatory subunit 5), PTGS2 (prostaglandin-endoperoxide synthase 2), RAP2B (RAP2B, member of RAS oncogene family), RASD1 (ras related dexamethasone induced 1), RGS16 (regulator of G protein signalling 16), TRAF6 (TNF receptor associated factor 6) and VEGFA (vascular endothelial growth factor A). The downregulated genes were IL4 (interleukin 4), MAPK13 (mitogen-activated protein kinase 13), MUC5B (mucin 5B, oligomeric mucus/gel-forming), TNFSF10 (TNF superfamily member 10) and TRAF5 (TNF receptor associated factor 5). For details see **Supplementary Tables 1**, **5** and **8**.

Supplementary Figure 5 Mucus secretion in the gills of rainbow trout exposed to the toxin-producing alga *Prymnesium parvum*. When exposed to UV-light, the two chloroplasts of *P. parvum* become fluorescent **(A, B)**. Only few algal cells tended to reach the gill lamellae **(C, D)**, while a relatively large number of algal cells was trapped in the secreted mucus **(E, F)**. Photos were taken with a Leica DM4008B-M microscope equipped with epifluorescence, using either transmitted white light **(A, C, E)** or UV-light of the same field **(B, D, F)**. Bars correspond to 10 µM **(A, B)** or 25 µM **(C–F)**.

## References

[1] Evans DH Piermarini PM Choe KP. The Multifunctional Fish Gill: Dominant Site of Gas Exchange, Osmoregulation, Acid-Base Regulation, and Excretion of Nitrogenous Waste. Physiol Rev (2005) 85:97–177. doi: 10.1152/physrev.00050.2003 15618479

[2] Koppang EO Kvellestad A Fischer U. “Fish Mucosal Immunity: Gill,”. In: Beck BH Peatman E, editors. Mucosal Health in Aquaculture, vol. . p . Amsterdam: Academic Press (2015). p. 93–133.

[3] Salinas I. The Mucosal Immune System of Teleost Fish. Biology (2015) 4:525–39. doi: 10.3390/biology4030525 PMC4588148 26274978

[4] Kleinow KM Nichols JW Hayton WL McKim JM Barron MG. “Toxicokinetics in Fishes,”. In: Di Giulio RT Hinton DE, editors. The Toxicology of Fishes, vol. . p . Boca Raton, FL: CRC Press (2008). p. 55–152.

[5] West AC Mizoro Y Wood SH Ince LM Iversen M Jørgensen EH. Immunologic Profiling of the Atlantic Salmon Gill by Single Nuclei Transcriptomics. Front Immunol (2021) 12:669889. doi: 10.3389/fimmu.2021.669889 34017342 PMC8129531

[6] Powell MD Harris JO Carson J Hill JV. Effects of Gill Abrasion and Experimental Infection With *Tenacibaculum Maritimum* on the Respiratory Physiology of Atlantic Salmon *Salmo Salar* Affected by Amoebic Gill Disease. Dis Aquat Org (2005) 63:169–74. doi: 10.3354/dao063169 15819432

[7] McIntyre JK Lundin JI Cameron JR Chow MI Davis JW Incardona JP. Interspecies Variation in the Susceptibility of Adult Pacific Salmon to Toxic Urban Stormwater Runoff. Environ Pollut (2018) 238:196–203. doi: 10.1016/j.envpol.2018.03 29554567

[8] Svendsen MBS Andersen NR Hansen PJ Steffensen JF. Effects of Harmful Algal Blooms on Fish: Insights From Prymnesium Parvum. Fishes (2018) 3:11. doi: 10.3390/fishes3010011

[9] Browning CL Green A Gray EP Hurt R Kane AB. Manganese Dioxide Nanosheets Induce Mitochondrial Toxicity in Fish Gill Epithelial Cells. Nanotoxicology (2021) 15:400–17. doi: 10.1080/17435390.2021.1874562 PMC8026737 33502918

[10] Clinton M Ferrier DEK Martin SAM Brierley AS. Impacts of Jellyfish on Marine Cage Aquaculture: An Overview of Existing Knowledge and the Challenges to Finfish Health. ICES J Mar Sci (2021) 78:1557–73. doi: 10.1093/icesjms/fsaa254

[11] Rességuier J Dalum AS Du Pasquier L Zhang YQ Koppang EO Boudinot P. Lymphoid Tissue in Teleost Gills: Variations on a Theme. Biology (2020) 9:127. doi: 10.3390/biology9060127 PMC7344468 32549335

[12] Salinas I Fernandez-Montero A Ding Y Sunyer JO. Mucosal Immunoglobulins of Teleost Fish: A Decade of Advances. Dev Comp Immunol (2021) 121:104079. doi: 10.1016/j.dci.2021.104079 33785432 PMC8177558

[13] Foyle KL Hess S Powell MD Herbert NA. What Is Gill Health and What Is Its Role in Marine Finfish Aquaculture in the Face of a Changing Climate? Front Mar Sci (2020) 7:400. doi: 10.3389/fmars.2020.00400

[14] Enders EC Boisclair D. Effects of Environmental Fluctuations on Fish Metabolism: Atlantic Salmon *Salmo Salar* as a Case Study. J Fish Biol (2016) 88:344–58. doi: 10.1111/jfb.12786 26577543

[15] Breitburg D Levin LA Oschlies A Grégoire M Chavez FP Conley DJ. Declining Oxygen in the Global Ocean and Coastal Waters. Science (2018) 359:46. doi: 10.1126/science.aam7240 29301986

[16] Nilsson GE. Gill Remodeling in Fish – A New Fashion or an Ancient Secret? J Exp Biol (2007) 210:2403–9. doi: 10.1242/jeb.000281 17601943

[17] Wood CM Eom J. The Osmorespiratory Compromise in the Fish Gill. Comp Biochem Physiol A Mol Integr Physiol (2016) 254:110895. doi: 10.1016/j.cbpa.2021.110895 33429056

[18] Pinsky ML Worm B Fogarty MJ Sarmiento JL Levin SA. Marine Taxa Track Local Climate Velocities. Science (2013) 341:1239–42. doi: 10.1126/science.1239352 24031017

[19] Moore SK Trainer VL Mantua NJ Parker MS Laws EA Backer LC. Impacts of Climate Variability and Future Climate Change on Harmful Algal Blooms and Human Health. Environ Health (2008) 7:S4. doi: 10.1186/1476-069X-7-S2-S4 PMC2586717 19025675

[20] Gobler CJ. Climate Change and Harmful Algal Blooms: Insights and Perspective. Harmful Algae (2020) 91:101731. doi: 10.1016/j.hal.2019.101731 32057341

[21] LeBlanc F Ditlecadet D Arseneau JR Steeves R Boston L Boudreau P. Isolation and Identification of a Novel Salmon Gill Poxvirus Variant From Atlantic Salmon in Eastern Canada. J Fish Dis (2019) 42:315–8. doi: 10.1111/jfd.12922 30511390

[22] Johnsen TM Eikrem W Olseng CD Tollefsen KE Bjerknes V. *Prymnesium Parvum*: The Norwegian Experience. JAWRA (2010) 46:6–13. doi: 10.1111/j.1752-1688.2009.00386.x

[23] Southard GM Fries LT Barkoh A. *Prymnesium Parvum*: The Texas Experience. JAWRA (2010) 46:14–23. doi: 10.1111/j.1752-1688.2009.00387.x

[24] Shilo M. Formation and Mode of Action of Algal Toxins. Bacteriol Rev (1967) 31:180–93. doi: 10.1128/br.31.3.180-193.1967 PMC378282 4864729

[25] Jonsson PR Pacvia H Toth G. Formation of Harmful Algal Blooms Cannot be Explained by Allelopathic Interactions. Proc Natl Acad Sci (2009) 106:11177–82. doi: 10.1073/pnas.0900964106 PMC2708709 19549831

[26] Remmel EJ Hambright KD. Toxin-Assisted Micropredation: Experimental Evidence Shows That Contact Micropredation Rather Than Exotoxicity is the Role of *Prymnesium* Toxins. Ecol Lett (2012) 15:126–32. doi: 10.1111/j.1461-0248.2011.01718.x 22132867

[27] Tillmann U. Kill and Eat Your Predator: A Winning Strategy of the Planktonic Flagellate Prymnesium Parvum. Aquat Microb Ecol (2003) 32:73–84. doi: 10.3354/ame032073

[28] Blossom HE Rasmussen SA Andersen NG Larsen TO Nielsen KF Hansen PJ. *Prymnesium Parvum* Revisited: Relationship Between Allelopathy, Ichthyotoxicity, and Chemical Profiles in 5 Strains. Aquat Toxicol (2014) 157:159–66. doi: 10.1016/j.aquatox.2014.10.006 25456230

[29] Manning SR La Claire JW. Prymnesins: Toxic Metabolites of the Golden Alga, *Prymnesium Parvum* Carter (Haptophyta). Mar Drugs (2010) 8:678–704. doi: 10.3390/md8030678 20411121 PMC2857367

[30] Binzer SB Svenssen DK Daugbjerg N Alves-de-Souza C Pinto E Hansen PJ. B- and C-Type Prymnesins Are Clade Specific Compounds and Chemotaxonomic Markers in Prymnesium Parvum. Harmful Algae (2019) 81:10–7. doi: 10.1016/j.hal.2018.11.010 30638493

[31] Taylor RB Hill BN Langan LM Chambliss CK Brooks BW. Sunlight Concurrently Reduces *Prymnesium Parvum* Elicited Acute Toxicity to Fish and Prymnesins. Chemosphere (2021) 263:127927. doi: 10.1016/j.chemosphere.2020.127927 32814137 PMC8117398

[32] Schug KA Skingel TR Spencer SE Serrano CA Le CQ Schug CA. Hemolysis, Fish Mortality, and LC-ESI-MS of Cultured Crude and Fractionated Golden Alga (*Prymnesium Parvum*). JAWRA (2010) 46:33–44. doi: 10.1111/j.1752-1688.2009.00389.x

[33] James SV Valenti TW Prosser KN Grover JP Roelke DL Brooks BW. Sunlight Amelioration of Prymnesium Parvum Acute Toxicity to Fish. J Plankton Res (2011) 33:265–72. doi: 10.1093/plankt/fbq082

[34] Bertin MJ Zimba PV Beauchesne KR Huncik KM Moeller PDR. Identification of Toxic Fatty Acid Amides Isolated From the Harmful Alga *Prymnesium Parvum* Carter. Harmful Algae (2012) 20:111–6. doi: 10.1016/j.hal.2012.08.005

[35] Grover JP Roelke DL Brooks BW Gable GM Neisch MT Hayden NJ. Ammonium Treatments to Suppress Toxic Blooms of *Prymnesium Parvum* in a Subtropical Lake of Semi-Arid Climate: Results From *in Situ* Mesocosm Experiments. Water Res (2013) 47:4274–85. doi: 10.1016/j.watres.2013.05.001 23764578

[36] Qin JL Hu ZX Zhang Q Xu N Yang YF. Toxic Effects and Mechanisms of *Prymnesium Parvum* (Haptophyta) Isolated From the Pearl River Estuary, China. Harmful Algae (2020) 96:101844. doi: 10.1016/j.hal.2020.101844 32560837

[37] Taylor RB Hill BN Bobbitt JM Hering AS Brooks BW Chambliss CK. Suspect and Non-Target Screening of Acutely Toxic Prymnesium Parvum. Sci Total Environ (2020) 715:136835. doi: 10.1016/j.scitotenv.2020.136835 32007880 PMC8080972

[38] Beszteri S Yang I Jaeckisch N Tillmann U Frickenhaus S Glockner G. Transcriptomic Response of the Toxic Prymnesiophyte *Prymnesium Parvum* (N. Carter) to Phosphorus and Nitrogen Starvation. Harmful Algae (2012) 18:1–15. doi: 10.1016/j.hal.2012.03.003

[39] Flood SL Burkholder JM. Imbalanced Nutrient Regimes Increase *Prymnesium Parvum* Resilience to Herbicide Exposure. Harmful Algae (2018) 75:57–74. doi: 10.1016/j.hal.2018.04.006 29778226

[40] Bergsson H Andersen NR Svendsen MBS Hansen PJ Steffensen JF. Respiratory Physiology of European Plaice (*Pleuronectes Platessa*) Exposed to Prymnesium Parvum. Fishes (2019) 4:32. doi: 10.3390/fishes4020032

[41] Dorantes-Aranda JJ Seger A Mardones JI Nichols PD Hallegraeff GM. Progress in Understanding Algal Bloom-Mediated Fish Kills: The Role of Superoxide Radicals, Phycotoxins and Fatty Acids. PloS One (2015) 10:e0133549. doi: 10.1371/journal.pone.0133549 26197230 PMC4509671

[42] Franco ME Hill BN Brooks BW Lavado R. *Prymnesium Parvum* Differentially Triggers Sublethal Fish Antioxidant Responses *In Vitro* Among Salinity and Nutrient Conditions. Aquat Toxicol (2019) 213:105214. doi: 10.1016/j.aquatox.2019.05.016 31185429

[43] Hill BN Saari GN Steele WB Corrales J Brooks BW. Nutrients and Salinity Influence *Prymnesium Parvum* (UTEX LB 2797) Elicited Sublethal Toxicity in *Pimephales Promelas* and Danio Rerio. Harmful Algae (2020) 93:101795. doi: 10.1016/j.hal.2020.101795 32307075 PMC8166212

[44] Andersen NG Lorenzen E Boutrup TS Hansen PJ Lorenzen N. Sublethal Concentrations of Ichthyotoxic Alga *Prymnesium Parvum* Affect Rainbow Trout Susceptibility to Viral Haemorrhagic Septicaemia Virus. Dis Aquat Org (2016) 117:187–95. doi: 10.3354/dao02946 26758652

[45] Martin SAM Dehler CE Król E. Transcriptomic Responses in the Fish Intestine. Dev Comp Immunol (2016) 64:103e117. doi: 10.1016/j.dci.2016.03.014 26995769

[46] Lindholm T Ohman P Kurki-Helasmo K Kincaid B Meriluoto J. Toxic Algae and Fish Mortality in a Brackish-Water Lake in Åland, SW Finland. Hydrobiologia (1999) 397:109–20. doi: 10.1023/A:1003667728458

[47] Castro R Jouneau L Tacchi L Macqueen DJ Alzaid A Secombes CJ. Disparate Developmental Patterns of Immune Responses to Bacterial and Viral Infections in Fish. Sci Rep (2015) 5:15458. doi: 10.1038/srep15458 26487553 PMC4614352

[48] Pooley NJ Tacchi L Secombes CJ Martin SA. Inflammatory Responses in Primary Muscle Cell Cultures in Atlantic Salmon (*Salmo Salar*). BMC Genomics (2013) 14:747. doi: 10.1186/1471-2164-14-747 24180744 PMC3819742

[49] Król E Douglas A Tocher DR Crampton VO Speakman JR Secombes CJ. Differential Responses of the Gut Transcriptome to Plant Protein Diets in Farmed Atlantic Salmon. BMC Genomics (2016) 17:156. doi: 10.1186/s12864-016-2473-0 26925977 PMC4772681

[50] Ritchie ME Phipson B Wu D Hu Y Law CW Shi W. Limma Powers Differential Expression Analyses for RNA-Sequencing and Microarray Studies. Nucleic Acids Res (2015) 43:e47. doi: 10.1093/nar/gkv007 25605792 PMC4402510

[51] R Core Team. R: A Language and Environment for Statistical Computing. In: R Foundation for Statistical Computing. Vienna, Austria (2018). Available at: https://www.R-project.org/.

[52] Song Y Salbu B Teien HC Heier LS Rosseland BO Høgåsen T. Hepatic Transcriptomic Profiling Reveals Early Toxicological Mechanisms of Uranium in Atlantic Salmon (*Salmo Salar*). BMC Genomics (2014) 15:694. doi: 10.1186/1471-2164-15-694 25145280 PMC4148957

[53] Król E Noguera P Shaw S Costelloe E Gajardo K Valdenegro V. Integration of Transcriptome, Gross Morphology and Histopathology in the Gill of Sea Farmed Atlantic Salmon (*Salmo Salar*): Lessons From Multi-Site Sampling. Front Genet (2020) 11:610. doi: 10.3389/fgene.2020.00610 32636874 PMC7316992

[54] Camacho C Coulouris G Avagyan V Ma N Papadopoulos J Bealer K. BLAST+: Architecture and Applications. BMC Bioinf (2009) 10:421. doi: 10.1186/1471-2105-10-421 PMC2803857 20003500

[55] Wells ML Karlson B Wulff A Kudela R Trick C Asnaghi V. Future HAB Science: Directions and Challenges in a Changing Climate. Harmful Algae (2020) 91:101632. doi: 10.1016/j.hal.2019.101632 32057342

[56] Bertin MJ Voronca DC Chapman RW Moeller PDR. The Effect of pH on the Toxicity of Fatty Acids and Fatty Acid Amides to Rainbow Trout Gill Cells. Aquat Toxicol (2014) 146:1–11. doi: 10.1016/j.aquatox.2013.09.026 24240104

[57] Anttila K Lewis M Prokkola JM Kanerva M Seppänen E Kolari I. Warm Acclimation and Oxygen Depletion Induce Species-Specific Responses in Salmonids. J Exp Biol (2015) 218:1471–7. doi: 10.1242/jeb.119115 25827840

[58] Kintner A Brierley AS. Cryptic Hydrozoan Blooms Pose Risks to Gill Health in Farmed North Atlantic Salmon (*Salmo Salar*). J Mar Biol Assoc UK (2019) 99:539–50. doi: 10.1017/s002531541800022x

[59] Metcalfe NB Van Leeuwen TE Killen SS. Does Individual Variation in Metabolic Phenotype Predict Fish Behaviour and Performance? J Fish Biol (2016) 88:298–321. doi: 10.1111/jfb.12699 26577442 PMC4991269

[60] Elias A Thrower F Nichols KM. Rainbow Trout Personality: Individual Behavioural Variation in Juvenile Oncorhynchus Mykiss. Behaviour (2018) 155:205–30. doi: 10.1163/1568539X-00003483

[61] Robertsen G Reid D Einum S Aronsen T Fleming IA Sundt-Hansen LE. Can Variation in Standard Metabolic Rate Explain Context-Dependent Performance of Farmed Atlantic Salmon Offspring? Ecol Evol (2019) 9:212–22. doi: 10.1002/ece3.4716 PMC6342125 30680108

[62] Montgomery MT Sajuthi SP Cho SH Everman JL Rios CL Goldfarbmuren KC. Genome-Wide Analysis Reveals Mucociliary Remodeling of the Nasal Airway Epithelium Induced by Urban Pm_2_. 5 Am J Respir Cell Mol Biol (2020) 63:172–84. doi: 10.1165/rcmb.2019-0454OC PMC7397762 32275839

[63] Bradner JE Hnisz D Young RA. Transcriptional Addiction in Cancer. Cell (2017) 168:629–43. doi: 10.1016/j.cell.2016.12.013 PMC5308559 28187285

[64] Cheedipudi SM Hu JZ Fan SY Yuan P Karmouch J Czernuszewicz G. Exercise Restores Dysregulated Gene Expression in a Mouse Model of Arrhythmogenic Cardiomyopathy. Cardiovasc Res (2020) 116:1199–213. doi: 10.1093/cvr/cvz199 PMC7177479 31350552

[65] Kragh ML Hansen LT. Initial Transcriptomic Response and Adaption of Listeria Monocytogenes to Desiccation on Food Grade Stainless Steel. Front Microbiol (2020) 10:3132. doi: 10.3389/fmicb.2019.03132 32038566 PMC6987299

[66] Wang Y Jia XZ Hsieh JCF Monson MS Zhang JB Shu DM. Transcriptome Response of Liver and Muscle in Heat-Stressed Laying Hens. Genes (2021) 12:255. doi: 10.3390/genes12020255 33578825 PMC7916550

[67] Lord JM Smith DC Roberts LM. Toxin Entry: How Bacterial Proteins Get Into Mammalian Cells. Cell Microbiol (1999) 1:85–91. doi: 10.1046/j.1462-5822.1999.00015.x 11207543

[68] Sandvig K van Deurs B. Delivery Into Cells: Lessons Learned From Plant and Bacterial Toxins. Gene Ther (2005) 12:865–72. doi: 10.1038/sj.gt.3302525 15815697

[69] Lahiani A Yavin E Lazarovici P. The Molecular Basis of Toxins’ Interactions With Intracellular Signaling *via* Discrete Portals. Toxins (2017) 9:107. doi: 10.3390/toxins9030107 PMC5371862 28300784

[70] Bhunia A Domadia PN Torres J Hallock KJ Ramamoorthy A Bhattacharjya S. NMR Structure of Pardaxin, a Pore-Forming Antimicrobial Peptide, in Lipopolysaccharide Micelles. J Biol Chem (2010) 285:3883–95. doi: 10.1074/jbc.M109.065672 PMC2823531 19959835

[71] Blanco J Acosta CP de la Puente MB Salgado C. Depuration and Anatomical Distribution of the Amnesic Shellfish Poisoning (ASP) Toxin Domoic Acid in the King Scallop Pecten Maximus. Aquat Toxicol (2002) 60:111–21. doi: 10.1016/S0166-445X(01)00274-0 12204591

[72] Bayne CJ Gerwick L. The Acute Phase Response and Innate Immunity of Fish. Dev Comp Immunol (2001) 25:725–43. doi: 10.1016/s0145-305x(01)00033-7 11602193

[73] Zou J Secombes CJ. The Function of Fish Cytokines. Biology (2016) 5:23. doi: 10.3390/biology5020023 PMC4929537 27231948

[74] Murakami M Kamimura D Hirano T. Pleiotropy and Specificity: Insights From the Interleukin 6 Family of Cytokines. Immunity (2019) 50:812–31. doi: 10.1016/j.immuni.2019.03.027 30995501

[75] Baggiolini M Clark-Lewis I. Interleukin-8, A Chemotactic and Inflammatory Cytokine. FEBS Lett (1992) 307:97–101. doi: 10.1016/0014-5793(92)80909-z 1639201

[76] Bernhard S Hug S Stratmann AEP Erber M Vidoni L Knapp CL. Interleukin 8 Elicits Rapid Physiological Changes in Neutrophils That Are Altered by Inflammatory Conditions. J Innate Immun (2021) 13:225–41. doi: 10.1159/000514885 PMC8460987 33857948

[77] Li XX Bechara R Zhao JJ McGeachy MJ Gaffen SL. IL-17 Receptor-Based Signaling and Implications for Disease. Nat Immunol (2019) 20:1594–602. doi: 10.1038/s41590-019-0514-y PMC6943935 31745337

[78] Yu JJ Gaffen SL. Interleukin-17: A Novel Inflammatory Cytokine That Bridges Innate and Adaptive Immunity. Front Biosci (2008) 13:170–7. doi: 10.2741/2667 17981535

[79] Coussens LM Werb Z. Inflammation and Cancer. Nature (2002) 420:860–7. doi: 10.1038/nature01322 PMC2803035 12490959

[80] Serhan CN Gupta SK Perretti M Godson C Brennan E Li YS. The Atlas of Inflammation Resolution (AIR). Mol Aspects Med (2020) 74:100894. doi: 10.1016/j.mam.2020.100894 32893032 PMC7733955

[81] Neurath MF. Resolution of Inflammation: From Basic Concepts to Clinical Application. Semin Immunopathol (2019) 41:627–31. doi: 10.1007/s00281-019-00771-2 31776733

[82] Timmermans S Souffriau J Libert C. A General Introduction to Glucocorticoid Biology. Front Immunol (2019) 10:1545. doi: 10.3389/fimmu.2019.01545 31333672 PMC6621919

[83] Oakley RH Cidlowski JA. The Biology of the Glucocorticoid Receptor: New Signaling Mechanisms in Health and Disease. J Allergy Clin Immunol (2013) 132:1033–44. doi: 10.1016/j.jaci.2013.09.007 PMC4084612 24084075

[84] Ouyang WJ Rutz S Crellin NK Valdez PA Hymowitz SG. Regulation and Functions of the IL-10 Family of Cytokines in Inflammation and Disease. Annu Rev Immunol (2011) 29:71–109. doi: 10.1146/annurev-immunol-031210-101312 21166540

[85] Ouyang WJ O’Garra A. IL-10 Family Cytokines IL-10 and IL-22: From Basic Science to Clinical Translation. Immunity (2019) 50:871–91. doi: 10.1016/j.immuni.2019.03.020 30995504

[86] Lawrence T. The Nuclear Factor NF-κb Pathway in Inflammation. Cold Spring Harb Perspect Biol (2009) 1:a001651. doi: 10.1101/cshperspect.a001651 20457564 PMC2882124

[87] Sanjabi S Zenewicz LA Kamanaka M Flavell RA. Anti- and Pro-Inflammatory Roles of TGF-β, IL-10, and IL-22 in Immunity and Autoimmunity. Curr Opin Pharmacol (2009) 9:447–53. doi: 10.1016/j.coph.2009.04.008 PMC2755239 19481975

[88] Barnabei L Laplantine E Mbongo W Rieux-Laucat F Weil R. NF-Kb: At the Borders of Autoimmunity and Inflammation. Front Immunol (2021) 12:716469. doi: 10.3389/fimmu.2021.716469 34434197 PMC8381650

[89] Dong C. Cytokine Regulation and Function in T Cells. Annu Rev Immunol (2021) 39:51–76. doi: 10.1146/annurev-immunol-061020-053702 33428453

[90] Levi M Keller TT van Gorp E ten Cate H. Infection and Inflammation and the Coagulation System. Cardiovasc Res (2003) 60:26–39. doi: 10.1016/S0008-6363(02)00857-X 14522404

[91] Wang M Wei JL Shang FT Zang K Ji T. Platelet-Derived Growth Factor B Attenuates Lethal Sepsis Through Inhibition of Inflammatory Responses. Int Immunopharmacol (2019) 75:105792. doi: 10.1016/j.intimp.2019.105792 31386981

[92] Tarnawski AS Ahluwalia A. The Critical Role of Growth Factors in Gastric Ulcer Healing: The Cellular and Molecular Mechanisms and Potential Clinical Implications. Cells (2021) 10:1964. doi: 10.3390/cells10081964 34440733 PMC8392882

[93] Sales CF dos Santos KPE Rizzo E Ribeiro RIMD dos Santos HB Thome RG. Proliferation, Survival and Cell Death in Fish Gills Remodeling: From Injury to Recovery. Fish Shellfish Immunol (2017) 68:10–8. doi: 10.1016/j.fsi.2017.07.001 28676337

[94] Piazzon MC Mladineo I Naya-Catala F Dirks RP Jong-Raadsen S Vrbatovic A. Acting Locally - Affecting Globally: RNA Sequencing of Gilthead Sea Bream With a Mild *Sparicotyle Chrysophrii* Infection Reveals Effects on Apoptosis, Immune and Hypoxia Related Genes. BMC Genomics (2019) 20:200. doi: 10.1186/s12864-019-5581-9 30866816 PMC6416957

[95] Rodger HD. Gill Disorders: An Emerging Problem for Farmed Atlantic Salmon (*Salmo Salar*) in the Marine Environment? Fish Vet J (2007) 9:38–48.

[96] Boerlage AS Ashby A Herrero A Reeves A Gunn GJ Rodger HD. Epidemiology of Marine Gill Diseases in Atlantic Salmon (*Salmo Salar*) Aquaculture: A Review. Rev Aquac (2020) 12:2140–59. doi: 10.1111/raq.12426

