# Supplementary figures and images for: Gill Transcriptomic Responses to Toxin-producing Alga Prymnesium parvum in Rainbow Trout
Source: Front Immunol. 2021 Dec 8;12:794593. doi: 10.3389/fimmu.2021.794593 (PMC8693183; doi:10.3389/fimmu.2021.794593)

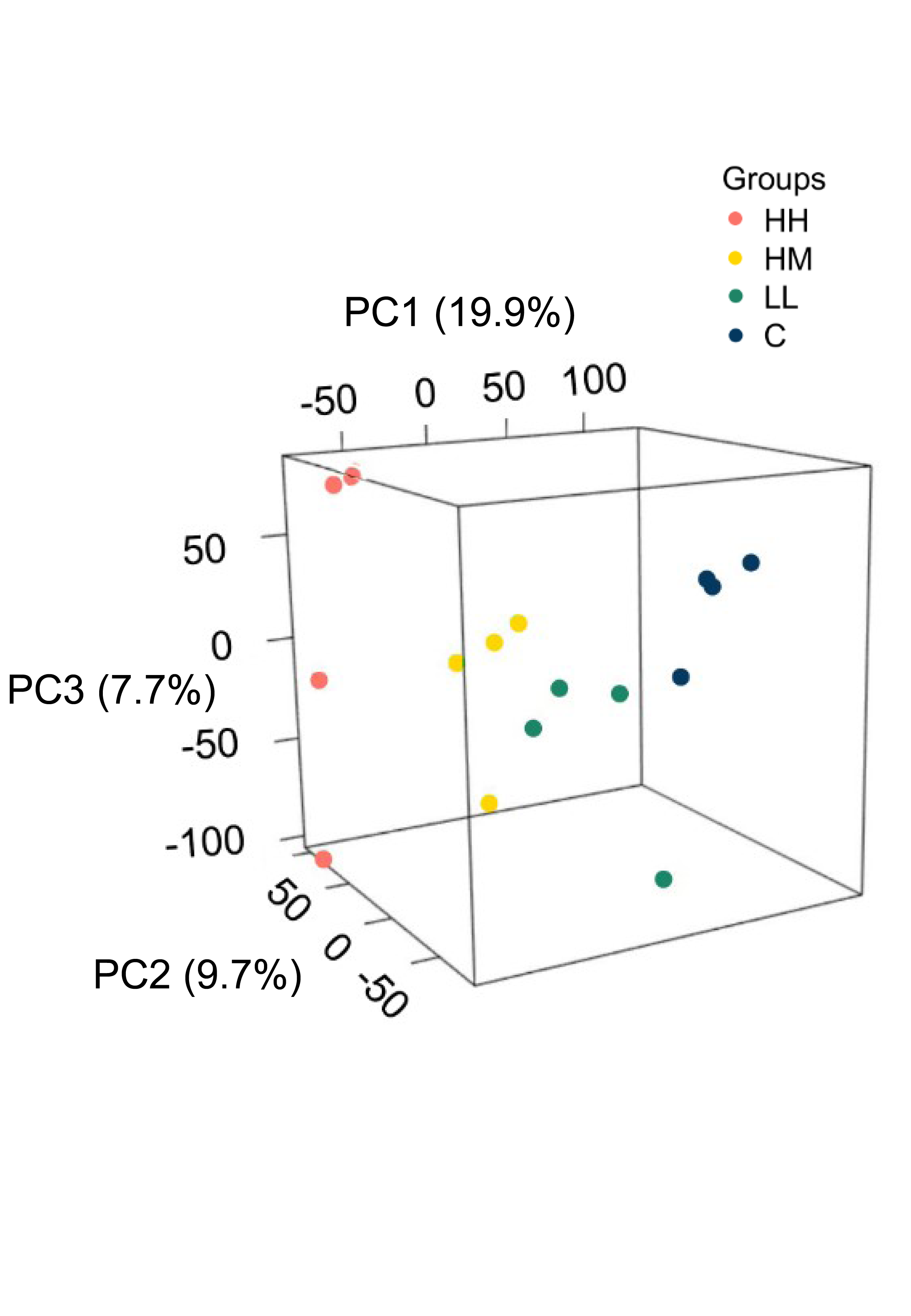

Supplement: Supplementary Figure 1 — 3D PCA (principal component analysis) plot of the gill transcriptome profiles in rainbow trout from HH (high exposure/high response), HM (high exposure/moderate response), LL (low exposure/low response) and C (control, no exposure/no response) groups. Each circle refers to all genes from one microarray hybridisation performed on 2–4 fish, with 4 hybridisations (biological replicates) per group (16 hybridisations in total). The percentage of total variance explained by PC1, PC2 and PC3 is given in parentheses. [file Image_1.tif]

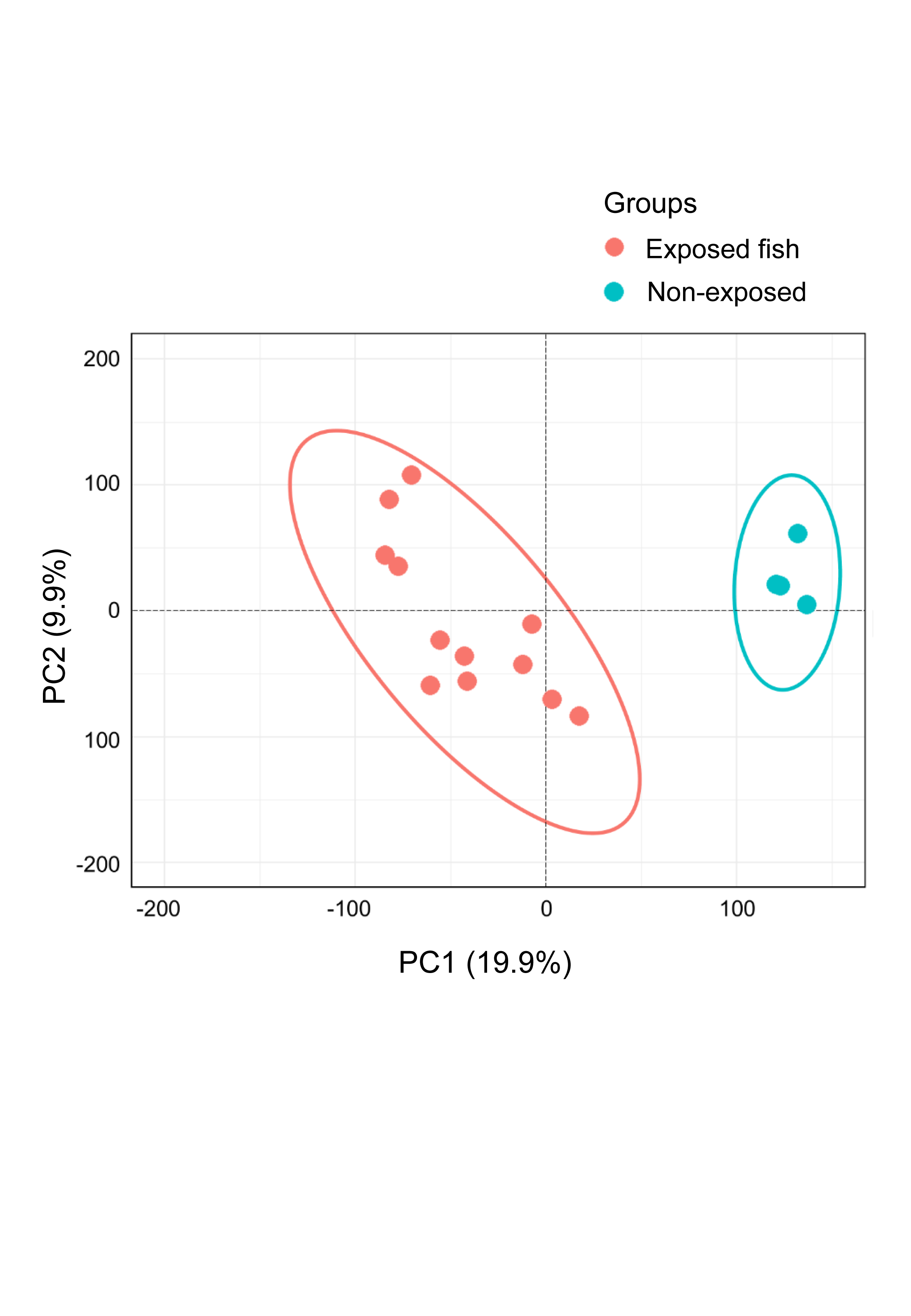

Supplement: Supplementary Figure 2 — Principal component analysis (PCA) of the gill transcriptome profiles in rainbow trout exposed to P. parvum (HH, HM and LL groups) and non-exposed control fish. Each circle refers to all genes from one microarray hybridisation performed on 2–4 fish, with 12 hybridisations representing the exposed fish and 4 hybridisations representing non-exposed fish. Ellipses indicate 95% confidence intervals. The percentage of total variance explained by PC1 and PC2 is given in parentheses. [file Image_2.tif]

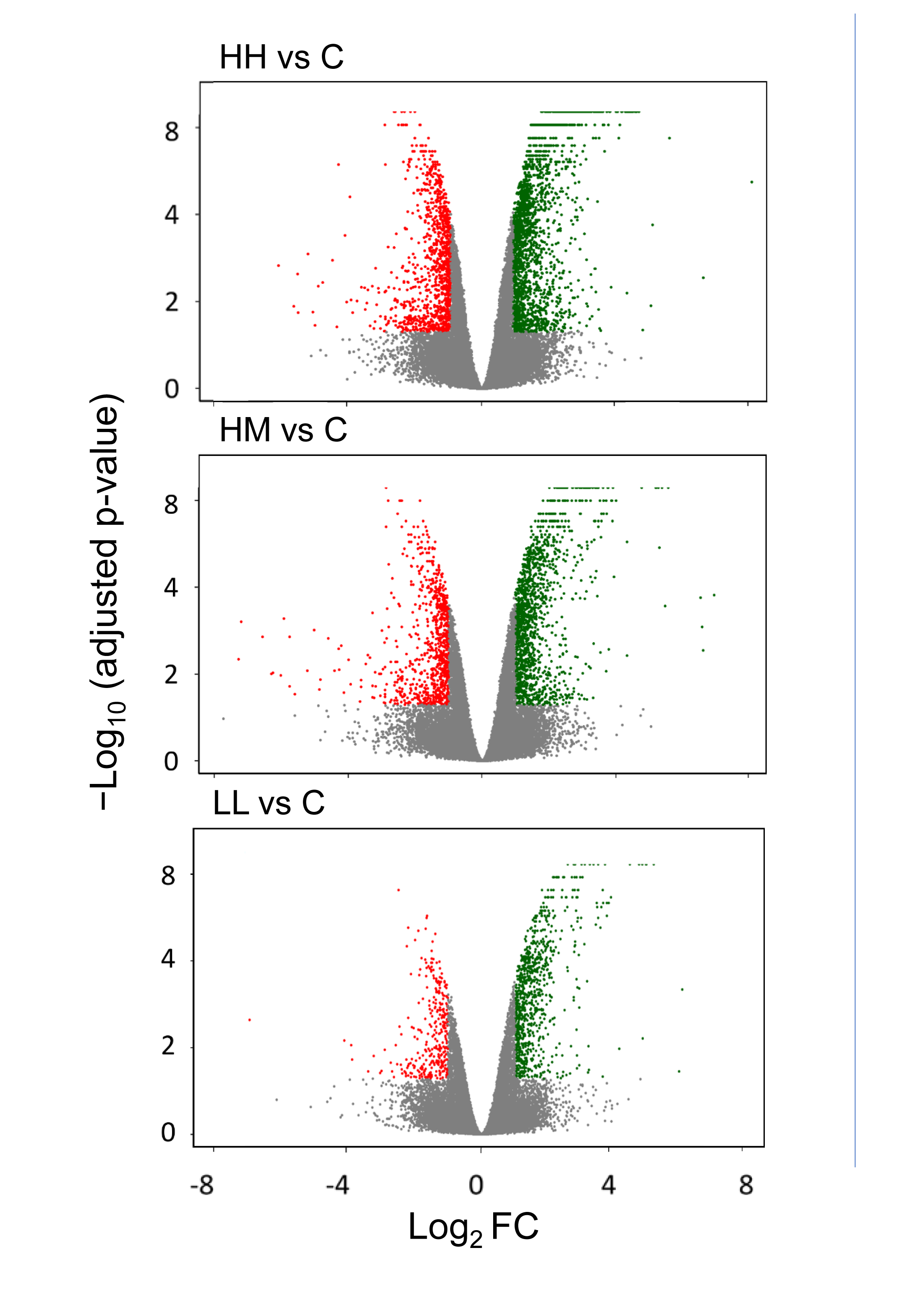

Supplement: Supplementary Figure 3 — Volcano plots of differential expression of RNA targets in the gill transcriptome of rainbow trout from HH (high exposure/high response), HM (high exposure/moderate response) and LL (low exposure/low response) groups in relation to C (control, no exposure/no response) group. Genes were considered differentially expressed at adjusted p-value < 0.05 and absolute Log2 FC > 1. Upregulated genes are in green, while downregulated genes are in red. [file Image_3.tif]

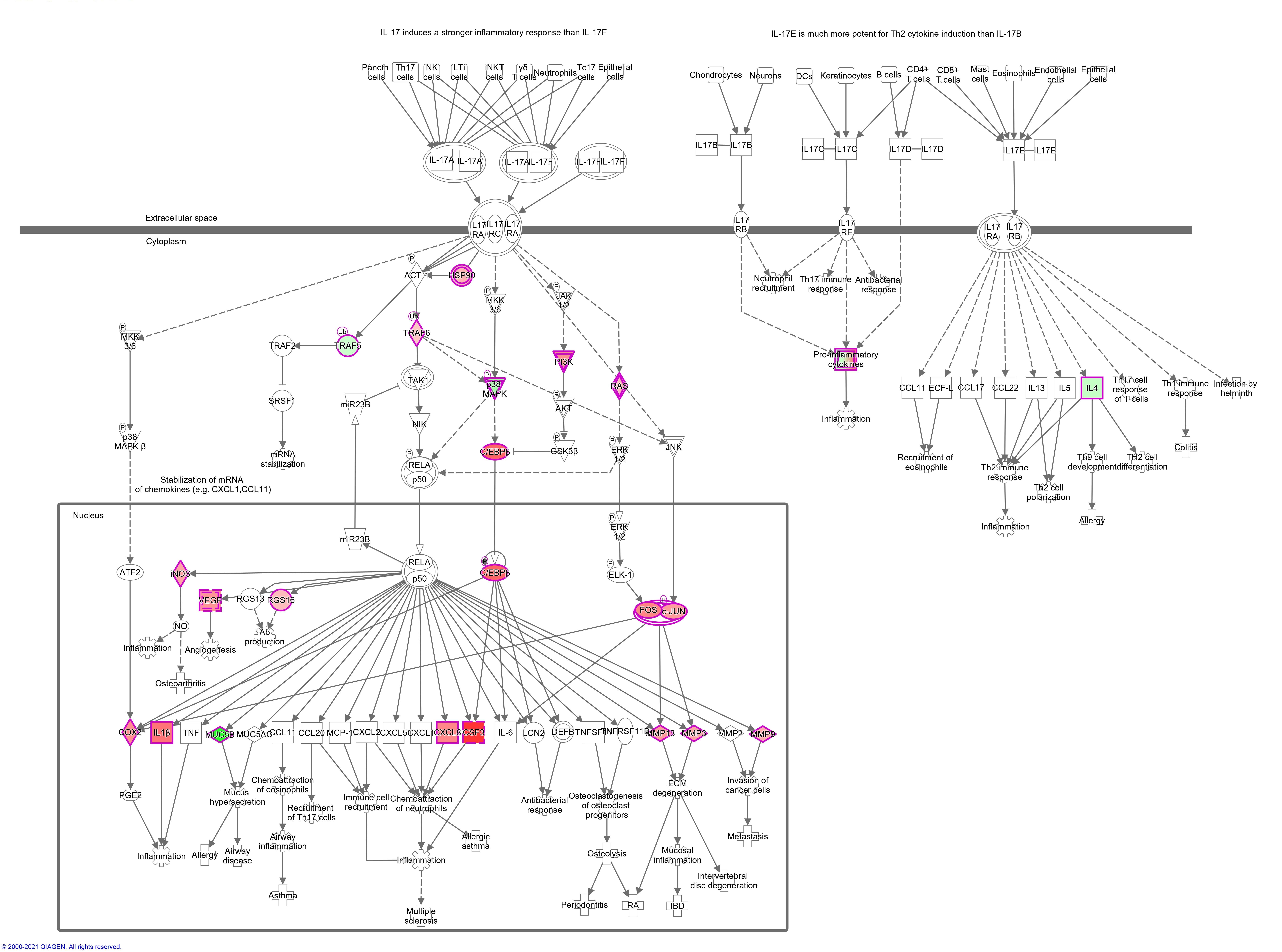

Supplement: Supplementary Figure 4 — Alterations of IL-17 Signalling pathway in the gill transcriptome of rainbow trout exposed to the high dose of P. parvum with high phenotypic response (HH group) in relation to the non-exposed control fish (C group). The pathway was identified as significant (Benjamini-Hochberg multiple testing correction p-value < 0.001) and activated (z-score ≥ 2) by Ingenuity Pathway Analysis (IPA). Among 187 genes that constitute the pathway, 28 were significantly altered by P. parvum exposure (yielding the gene ratio of 0.150), including 23 genes upregulated (in red) and 5 genes downregulated (in green). The upregulated genes were CEBPB (CCAAT enhancer binding protein beta), CSF3 (colony stimulating factor 3), FOS (Fos proto-oncogene, AP-1 transcription factor subunit), HRAS (HRas proto-oncogene, GTPase), HSP90AA1 (heat shock protein 90 alpha family class A member 1), HSP90AB1 (heat shock protein 90 alpha family class B member 1), IL11 (interleukin 11), IL12B (interleukin 12B), IL1B (interleukin 1 beta), IL8/CXCL8 (C-X-C motif chemokine ligand 8), JUN (Jun proto-oncogene, AP-1 transcription factor subunit), LTB (lymphotoxin beta), MMP13 (matrix metallopeptidase 13), MMP3 (matrix metallopeptidase 3), MMP9 (matrix metallopeptidase 9), NOS2 (nitric oxide synthase 2), PIK3R5 (phosphoinositide-3-kinase regulatory subunit 5), PTGS2 (prostaglandin-endoperoxide synthase 2), RAP2B (RAP2B, member of RAS oncogene family), RASD1 (ras related dexamethasone induced 1), RGS16 (regulator of G protein signalling 16), TRAF6 (TNF receptor associated factor 6) and VEGFA (vascular endothelial growth factor A). The downregulated genes were IL4 (interleukin 4), MAPK13 (mitogen-activated protein kinase 13), MUC5B (mucin 5B, oligomeric mucus/gel-forming), TNFSF10 (TNF superfamily member 10) and TRAF5 (TNF receptor associated factor 5). For details see Supplementary Tables 1, 5 and 8. [file Image_4.tif]

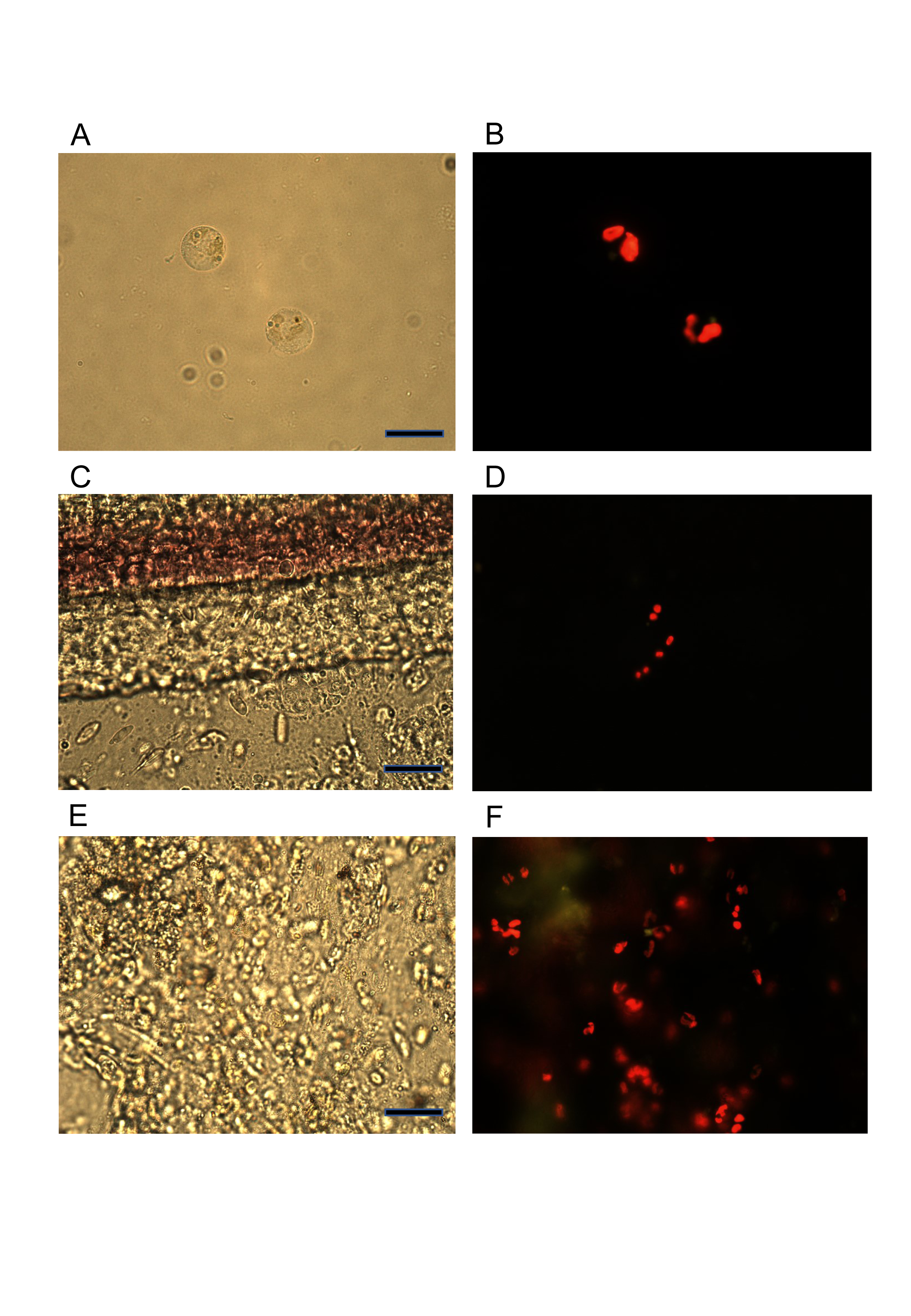

Supplement: Supplementary Figure 5 — Mucus secretion in the gills of rainbow trout exposed to the toxin-producing alga Prymnesium parvum. When exposed to UV-light, the two chloroplasts of P. parvum become fluorescent (A, B). Only few algal cells tended to reach the gill lamellae (C, D), while a relatively large number of algal cells was trapped in the secreted mucus (E, F). Photos were taken with a Leica DM4008B-M microscope equipped with epifluorescence, using either transmitted white light (A, C, E) or UV-light of the same field (B, D, F). Bars correspond to 10 µM (A, B) or 25 µM (C–F). [file Image_5.tif]
